# Molecular Characterization of Gβ-Like Protein CpcB Involved in Antifungal Drug Susceptibility and Virulence in *A. fumigatus*

**DOI:** 10.3389/fmicb.2016.00106

**Published:** 2016-02-09

**Authors:** Zhendong Cai, Yanfei Chai, Caiyun Zhang, Ruoyun Feng, Hong Sang, Ling Lu

**Affiliations:** ^1^Jiangsu Key Laboratory for Microbes and Functional Genomics, Jiangsu Engineering and Technology Research Center for Microbiology, College of Life Sciences, Nanjing Normal UniversityNanjing, China; ^2^Department of Dermatology, Jinling Hospital, School of Medicine, Nanjing UniversityNanjing, China

**Keywords:** Gβ-like CpcB, G protein, drug susceptibility, virulence, *Aspergillus fumigatus*

## Abstract

*Aspergillus fumigatus* is an airborne human fungal pathogen that can survive in a wide range of environmental condition. G protein complex transduces external signals from a variety of stimuli outside a cell to its interior effectors in all eukaryotes. Gβ-like CpcB (cross pathway control B) belongs to a WD40 repeat protein family with the conserved G–H and W–D residues. Previous studies have demonstrated that Gβ-like proteins cooperate with related signal transduction proteins to function during many important developmental processes in *A. fumigatus*. However, the molecular characteristics of Gβ-like CpcB have not yet been identified. In this study, we demonstrated that the G–H residues in WD repeat 1, 2, 3, and the W–D residue in WD repeat 2 of CpcB are required not only to control normal hyphal growth and conidiation but also to affect antifungal drug susceptibility. The enhanced drug resistance might be due to reduced intracellular drug accumulation and altered ergosterol component. Moreover, we find that the first G–H residue of CpcB plays an important role in the virulence of *A. fumigatus*. To our knowledge, this is the first report for finding the importance of the conserved G–H and W–D residues for a Gβ-like protein in understanding of G protein functions.

## Introduction

*Aspergillus fumigatus* is a saprophytic fungus with a large number of buoyant airborne conidia, and it plays an essential role in carbon and nitrogen recycling due to its characteristic metabolic ability to assimilate organic carbon and some non-elemental sources of nitrogen (Tekaia and Latge, 2005; Lapp et al., 2014). Moreover, thermo-tolerance enables it to survive in a broad range of environmental conditions and, accordingly, to be abundant in organic debris in soil and decaying vegetation (Haines, 1995; Seyedmousavi et al., 2015). Compared with other *Aspergilli*, the conidia of *A. fumigatus* that are released into the atmosphere have a diameter that is small enough (2–3 μm) to penetrate deep into the lung alveoli, which may contribute to the pathogenicity of this fungus (Latgé, 1999; Brookman and Denning, 2000). As an important opportunistic pathogen, *A. fumigatus* has become the most prevalent airborne fungal pathogen, causing severe and usually fatal invasive infections in immunocompromised hosts, especially individuals with cancer, leukemia, AIDS, organ transplantation, and chronic granulomatous disease (CGD) (Dagenais and Keller, 2009; Hillmann et al., 2015; Throckmorton et al., 2015). Previous studies have demonstrated that virulence determinants of *A. fumigatus* are multifactorial and are associated with the biological characteristics of the fungus and the immune status of the individual. The virulence-related genes can be classified according to the process they are associated with, such as thermotolerance, cell wall integrity, toxins, nutrient uptake during invasive growth, stress response, and allergens (Abad et al., 2010). In addition, proteins involved in calcium signaling and G protein signaling, which play important roles in detecting and transmitting environmental signals, have been shown to be important for virulence (Abad et al., 2010).

WD repeat proteins are members of a large protein family with a common beta-propeller structure based on the presence of four or more repeating units. The WD repeat (also known as the WD40 or β-transducin repeat) is composed of a 44–60-long sequence of residues with the Gly-His (G–H) dipeptide at the N-terminus and the Trp-Asp (W–D) dipeptide at the C-terminus, often terminating in a W–D dipeptide. Neither the G–H nor the W–D dipeptide is absolutely conserved with the WD repeats exhibiting a variable length in the N-terminal and C-terminal regions (Neer et al., 1994; Smith et al., 1999). Thus, they are found in all eukaryotes and are linked to a wide variety of functions such as signal transduction, transcriptional regulation, cell cycle control and apoptosis (Neer et al., 1994; Smith et al., 1999). Moreover, WD proteins are involved in fungal virulence, such as FSR1 (*Fusarium* stalk rot 1) in *Fusarium verticillioides* and *F. graminearum*, and Rak1 (mammalian RACK1 homolog) in *Ustilago maydis* (Shim et al., 2006; Wang et al., 2011). In addition, a previous study demonstrated that histidine of G–H residues and tryptophan of W–D residues are important for hydrogen bonding and for stabilizing the toroidal structure (Sondek et al., 1996), which may be broadly applicable to conserved WD repeat proteins across fungal species.

Among the WD repeat proteins, the Gβ subunit of heterotrimeric G proteins, which functions as a tight dimer with Gγ subunit (Gβγ), is one of the most well characterized WD repeat proteins with a known three-dimensional structure (Sondek et al., 1996; Downes and Gautam, 1999; Smith et al., 1999). To date, it has been found that mammalian genomes encoding multiple forms of WD repeat proteins possess six characterized Gβ subunits, while the genome of the yeast *Saccharomyces cerevisae* containing twelve seven-WD-repeat proteins encodes one Gβ subunit and one Gβ-like protein Asc1/Cpc2 (Whiteway et al., 1989; Smith et al., 1999; Valerius et al., 2007). CpcB, a homolog of Asc1, is a Gβ-like protein and belongs to a family of WD40 repeat proteins in *A. fumigatus.* Based on bioinformatics analyses, CpcB is highly conserved in eukaryotes with Gβ-like homologs exhibiting identities ranging from 60.4 to 95.6% (Cai et al., 2015). Previous studies have demonstrated that CpcB plays important roles in hyphal growth and conidiation in *A. fumigatus* (Kong et al., 2013; Cai et al., 2015). Despite its important functions, the molecular characterization of CpcB has not been performed. In the present study, using molecular techniques and phenotype analysis, we verified the function of the conserved G–H and W–D residues of CpcB. Based on the transcriptome analysis and drug susceptibility testing, we demonstrated that Gβ-like CpcB is involved in the maintenance of normal antifungal drug susceptibility, which is associated with the functions of necessary G–H and W–D residues. Moreover, we validated the relevance of the virulence of selected G–H residue mutant through virulence testing of the *cpcB* mutants. To our knowledge, this is the first study to demonstrate the important functions of conserved G–H and W–D residues in Gβ-like homologs.

## Materials and Methods

### Strains, Oligonucleotides, Media, and Transformation

A list of all *A. fumigatus* strains and oligonucleotides used in this study is provided in **Table 1** and **Supplementary Table 1**, respectively. Parental wild-type (WT), *ΔcpcB* mutant and *cpcB*-reconstituted strains used in the present study were identical to those referred to as A1160C’, CZ01, and CZ02, respectively (Jiang et al., 2014; Cai et al., 2015). All colonies of the *A. fumigatus* strains were grown on YAG, which contains 2% glucose, 0.5% yeast extract, trace elements, and 2% agar. For the YG liquid medium, the agar was removed from the YAG. Mycelia were harvested from liquid Minimal Medium (MM), which contains 1% glucose, trace elements, and 50 ml L^-1^ 20 × salt. Transformation was performed according to a previously described method (Osmani et al., 1988; May, 1989).

**Table 1:** *Aspergillus fumigatus* strains used in this study.

Strain	Genotype	Reference
A1160C’	*Δku80*;A1160::*pyrG*	Jiang et al., 2014
CZ01	*Δku80*; *pyrG*; *ΔcpcB*::*pyr4*	Cai et al., 2015
CZ02	*Δku80*; *pyrG*; *ΔcpcB*::*pyr4*; *cpcB* (p)::*cpcB*	Cai et al., 2015
CZA01	*Δku80*; *pyrG*; *ΔcpcB*::*pyr4*; *cpcB* (p)::*cpcB*(ΔN)	This study
CZA02	*Δku80*; *pyrG*; *ΔcpcB*::*pyr4*; *cpcB* (p)::*cpcB*(ΔWD2-4)	This study
CZA03	*Δku80*; *pyrG*; *ΔcpcB*::*pyr4*; *cpcB* (p)::*cpcB*(ΔWD5-6)	This study
CZA04	*Δku80*; *pyrG*; *ΔcpcB*::*pyr4*; *cpcB* (p)::*cpcB*(ΔC)	This study
CZA01-GFP	*Δku80*; *pyrG*; *ΔcpcB*::*pyr4*; *cpcB* (p)::*cpcB*(ΔN)::GFP	This study
CZA02-GFP	*Δku80*; *pyrG*; *ΔcpcB*::*pyr4*; *cpcB* (p)::*cpcB*(ΔWD2-4)::GFP	This study
CZA03-GFP	*Δku80*; *pyrG*; *ΔcpcB*::*pyr4*; *cpcB* (p)::*cpcB*(ΔWD5-6)::GFP	This study
CZA04-GFP	*Δku80*; *pyrG*; *ΔcpcB*::*pyr4*; *cpcB* (p)::*cpcB*(ΔC)::GFP	This study
CZA05	*Δku80*; *pyrG*; *ΔcpcB*::*pyr4*; *cpcB* (p)::*cpcB*^(G13A,H14E)^	This study
CZA06	*Δku80*; *pyrG*; *ΔcpcB*::*pyr4*; *cpcB* (p)::*cpcB*^(G61A,H62E)^	This study
CZA07	*Δku80*; *pyrG*; *ΔcpcB*::*pyr4*; *cpcB* (p)::*cpcB*^(G103A,H104E)^	This study
CZA08	*Δku80*; *pyrG*; *ΔcpcB*::*pyr4*; *cpcB* (p)::*cpcB*^(G146A,H147E)^	This study
CZA09	*Δku80*; *pyrG*; *ΔcpcB*::*pyr4*; *cpcB* (p)::*cpcB*^(G190A,H191E)^	This study
CZA10	*Δku80*; *pyrG*; *ΔcpcB*::*pyr4*; *cpcB* (p)::*cpcB*^(W83R,D84K)^	This study
CZA11	*Δku80*; *pyrG*; *ΔcpcB*::*pyr4*; *cpcB* (p)::*cpcB*^(W170R,D171K)^	This study
CZA12	*Δku80*; *pyrG*; *ΔcpcB*::*pyr4*; *cpcB* (p)::*cpcB*^(W219R,D220K)^	This study


### Construction of the Plasmids and Strains for the GFP-Labeled Truncation Experiment for CpcB

To construct the specific CpcB-GFP plasmids with the truncated region of CpcB shown in **Figure 2A**, we first generated the CpcB-GFP plasmid (pCA01) embedded with a full-length CpcB fused to the GFP gene as follows. The 5′ flanking region (3030 bp, the *cpcB* promoter sequences and the full-length *cpcB* ORF without the stop codon) and the 3′ flanking region (739 bp, 3UTR) were amplified from *A. fumigatus* A1160 genomic DNA (gDNA) with the primer pairs CZ-P01/CZ-P07 and CZ-P05/CZ-P08, respectively. A 752-bp GFP fragment was amplified from plasmid pFNO3 using the primers GFP-F and GFP-R. The three PCR products were fused using the nested primer pair CZ-P02/CZ-P03 to generate a fusion fragment (4094 bp). As a selectable marker, a 4269-bp hygromycin fragment (*hygB*) was amplified from plasmid pAN7-1 with the primers Hyg-F and Hyg-R. The final 8120-bp PCR products were generated by fusion PCR using the primers CZ-P04 and CZ-P06, and then cloned using the pEASY-Blunt Cloning Kit (TransGen Biotech) to generate plasmid pCA01.

The CpcB-GFP reconstruction plasmid pCA02 (ΔN) carrying the N-terminal deletion of CpcB–GFP was generated as follows. Using plasmid pCA01 as the template, 1397-bp and 6453-bp DNA fragments were amplified with the primer pairs CZ-P06/N-R and CZ-P04/N-F, respectively. The final 7850-kb DNA fragment was generated from these two DNA fragments through fusion PCR using the primers CZ-P04 and CZ-P06. Next, the fragment was cloned using the pEASY-Blunt Cloning Kit (TransGen Biotech) to generate plasmid pCA02 (ΔN). A similar strategy was used to construct plasmids containing CpcB-GFP with deletions of WD repeat 2–4, WD repeat 5–6 and the C-terminal truncation excluding the stop codon, which were denoted pCA03 (ΔWD2-4), pCA04 (ΔWD5-6), and pCA05 (ΔC), respectively. The GFP-labeled strains of CZA01-GFP (ΔN), CZA02-GFP (ΔWD2-4), CZA03-GFP (ΔWD5-6), and CZA04-GFP (ΔC) were generated by transforming plasmids pCA02 (ΔN), pCA03 (ΔWD2-4), pCA04 (ΔWD5-6), and pCA05 (ΔC), respectively, into the *ΔcpcB* mutant. The transformants were screened on MM containing 150 μg/ml hygromycin B.

### Immunoblotting Experiment

As previously described (Zhong et al., 2014; Cai et al., 2015) for protein extraction from *A. fumigatus* mycelia, conidia were inoculated into liquid MM and shaken at 220 rpm on a rotary shaker at 37°C for 20 h. The tissue was ground in liquid nitrogen and rapidly suspended in ice-cold extraction buffer [50 mM HEPES (pH 7.4), 137 mM KCl, 10% glycerol, 1 μg/ml pepstatin A, 1 μg/ml leupeptin, 1 mM PMSF]. Equal amounts of protein (40 μg) per lane were subjected to 10% SDS-PAGE and then transferred to a polyvinylidene difluoride (PVDF) membrane (Immobilon-P; Millipore) in 384 mM glycine, 50 mM Tris (pH 8.4), and 20% methanol at 250 mA for 1.5 h. The membrane was blocked with phosphate-buffered saline (PBS) containing 5% milk and 0.1% Tween 20. Next, the membrane was probed sequentially with a 1:3,000 dilution of anti-GFP antibody (Roche Applied Science) and horseradish peroxidase-conjugated goat anti-rabbit IgG diluted in PBS containing 5% milk and 0.1% Tween 20. The blot was developed using enhanced chemiluminescence (ECL; Amersham).

### Construction of Plasmids and Strains for the CpcB Truncation Experiment

The CpcB reconstruction plasmid pCA06 (ΔN) with the deletion in the N-terminus of CpcB was generated as follows. Using gDNA as the template, 1589-bp and 1911-bp DNA fragments were amplified with the primer pairs CZ-P01/N-R and CZ-P05/N-F, respectively. The two PCR products were fused together with primers CZ-S and CZ-A. The resulting 3053-bp DNA fragment containing the promoter sequence, truncated ORF and 3′UTR was cloned into plasmid pAN7-1 using the ClonExpress II One Step Cloning Kit (Vazyme^TM^, C112-02) to generate plasmid pCA06 (ΔN). A similar strategy was used to construct the plasmids with deletions of WD repeat 2–4, WD repeat 5–6, or the C-terminal truncation excluding the stop codon, which were referred to as pCA07 (ΔWD2-4), pCA08 (ΔWD5-6), and pCA09 (ΔC), respectively. The truncation strains CZA01 (ΔN), CZA02 (ΔWD2-4), CZA03 (ΔWD5-6), and CZA04 (ΔC) were generated by transforming the respective related plasmids into the *ΔcpcB* mutant. The transformants were screened on MM containing 150 μg/ml hygromycin B.

### Construction of Plasmids and Strains for the CpcB Point Mutation Experiment

The CpcB reconstruction plasmid pCA10 carrying a mutation in the first G–H residue in WD repeat 1 was constructed as follows. Using gDNA as the template, 1616-bp and 2154-bp DNA fragments were amplified with primer pairs CZ-P01/1 G–H–R and CZ-P05/1 G–H–F, respectively. The two PCR products were fused together with primers CZ-S and CZ-A. The resulting 3323-bp DNA fragment (the complete ORF harboring a G–H mutation, promoter sequence and 3′UTR) was cloned into plasmid pAN7-1 using the ClonExpress II One Step Cloning Kit (Vazyme^TM^, C112-02) to generate plasmid pCA10 (1 G–H mutation). A similar strategy was used to construct the plasmids carrying mutations in the second to the fifth G–H residues and the first to the third W–D residues, which were referred to as pCA11 (2 G–H mutation), pCA12 (3 G–H mutation), pCA13 (4 G–H mutation), pCA14 (5 G–H mutation), pCA15 (1 W–D mutation), pCA16 (2 W–D mutation), and pCA17 (3 W–D mutation), respectively. The strains containing point mutation of the first to the fifth G–H residues, CZA05-CZA09, and the first to the third W–D residues, CZA10–CZA12, were generated by transforming the plasmids for the G–H mutation pCA10–pCA14 and the W–D mutation pCA15–pCA17 into the *ΔcpcB* mutant, respectively. The transformants were screened on MM containing 150 μg/ml hygromycin B.

### Measurement of R6G Uptake and Glucose-Induced Efflux

Intracellular R6G was evaluated using a previously described protocol (Maesaki et al., 1999) with some modifications. Briefly, conidia were inoculated into YG medium at approximately 5 × 10^6^ fresh conidia/ml and allowed to germinate for approximately 5 h. The conidia were then harvested and centrifuged at 9000 *g* for 2 min. The pellets were washed twice with glucose-free PBS, and re-suspended in glucose-free PBS at a concentration of 5 × 10^6^ conidia/ml. To assess the uptake of R6G, R6G was added to the conidial suspension at a final concentration of 10 μM and incubated for 1 h at 37 °C. To remove extracellular R6G, 1 ml of the equilibrated cells were washed and re-suspended in 1 ml of glucose-free PBS buffer. The absorption of the resulting cell suspension (1 ml) was measured at 527 nm. To assess energy-dependent efflux, R6G was added to the conidial suspension at a final concentration of 10 μM and incubated for 1 h at 37°C. Next, 0.1 g/ml of glucose was added to the sample, followed by an incubation for 0.5 h at 37 °C. One milliliter of the equilibrated cells were then washed and re-suspended in 1 ml of glucose-free PBS buffer to measure the absorption. The flow cytometric analysis was performed using an Accuri C6 instrument and analyzed with BD Accuri C6 software (BD Biosciences).

### MIC Assay

Equal number of conidia (4 × 10^3^) from the parental WT, *ΔcpcB*, 1 G–H mutant strains were cultured in a 96-well plate at 37°C for 24 h in YG liquid medium without or with drugs at three indicated concentrations, respectively. O.D value was detected at 530 nm by SpectraMax M2 system and data were analyzed with the SoftMax Pro v5.0.1 software package.

### Total Ergosterol Extraction

Total ergosterol in the *A. fumigatus* strains was extracted using a previously described protocol (Alcazar-Fuoli et al., 2008). Briefly, mycelia were cultured for 18 h in 100 ml of liquid MM media. Subsequently, they were harvested, dried and ground to a fine powder. Two hundred milligrams of the ground mycelia were treated with 3 ml of 25% alcohol potassium hydroxide solution (3:2 methanol:ethanol) and mixed by vortexing for 1 min. After the mixture was incubated in an 85 °C water bath for 1 h, the ergosterol was treated with 1 ml of sterile distilled water and 3 ml of pentane followed by vigorous vortexing for 3 min. The upper pentane layer was transferred to a clean glass tube and evaporated in a fume hood at room temperature. The dried-down samples were re-dissolved in 1 ml of methanol and syringe-filtered through 0.2 μm-pore-size filters. Total ergosterol was analyzed using HPLC (Agilent Technologies) and detected at 280 nm using an AQ-C18 column (250 mm by 4.6 mm, 5 μm). Elution was conducted at a flow rate of 1 ml min^-1^ with a mobile phase containing water and methanol eluent (100% HPLC grade).

### Virulence Test

Animal infection experiments were conducted following the guidelines and approved protocols of the Jiangsu Province Experiment Animal Care and Use Committee. Using an immunosuppressed murine model, the virulence assay was performed according to similar approaches described previously (Cai et al., 2015). White male ICR mice (6 weeks old, 20–22 g) were injected intraperitoneally with cyclophosphamide at 150 mg/kg of body weight on days-3 and -1, followed by 75 mg/kg of body weight on days 3, 6, and 9. In addition to cyclophosphamide, hydrocortisone acetate was also administered to immunosuppressed mice via subcutaneous injection at 40 mg/kg of body weight only on day-1. And then mice were anesthetized with pelltobarbitalum natricum by intraperitoneal injection for minutes on day 0. In 15 mice in each group, 30 μl of saline containing 2.5 × 10^6^ conidia from the parental WT or the relative *cpcB* mutant strains were inoculated into the mice of the respective group via endotracheal intubation; the saline group received 30 μl of saline without conidia as the control. The mice were maintained in sterile conditions and fed with sterilized water containing tetracycline (1 mg/ml, Sigma) to prevent bacterial infection. The survival rate was monitored daily until day 10 after inoculation. Lungs were removed from the dead animals and maintained in 4% formaldehyde for fixation before periodic acid–Schiff staining, according to a standard procedure (Schmalhorst et al., 2008).

## Results

### Structural Features of the WD40 Repeat Protein CpcB

**FIGURE 1:**
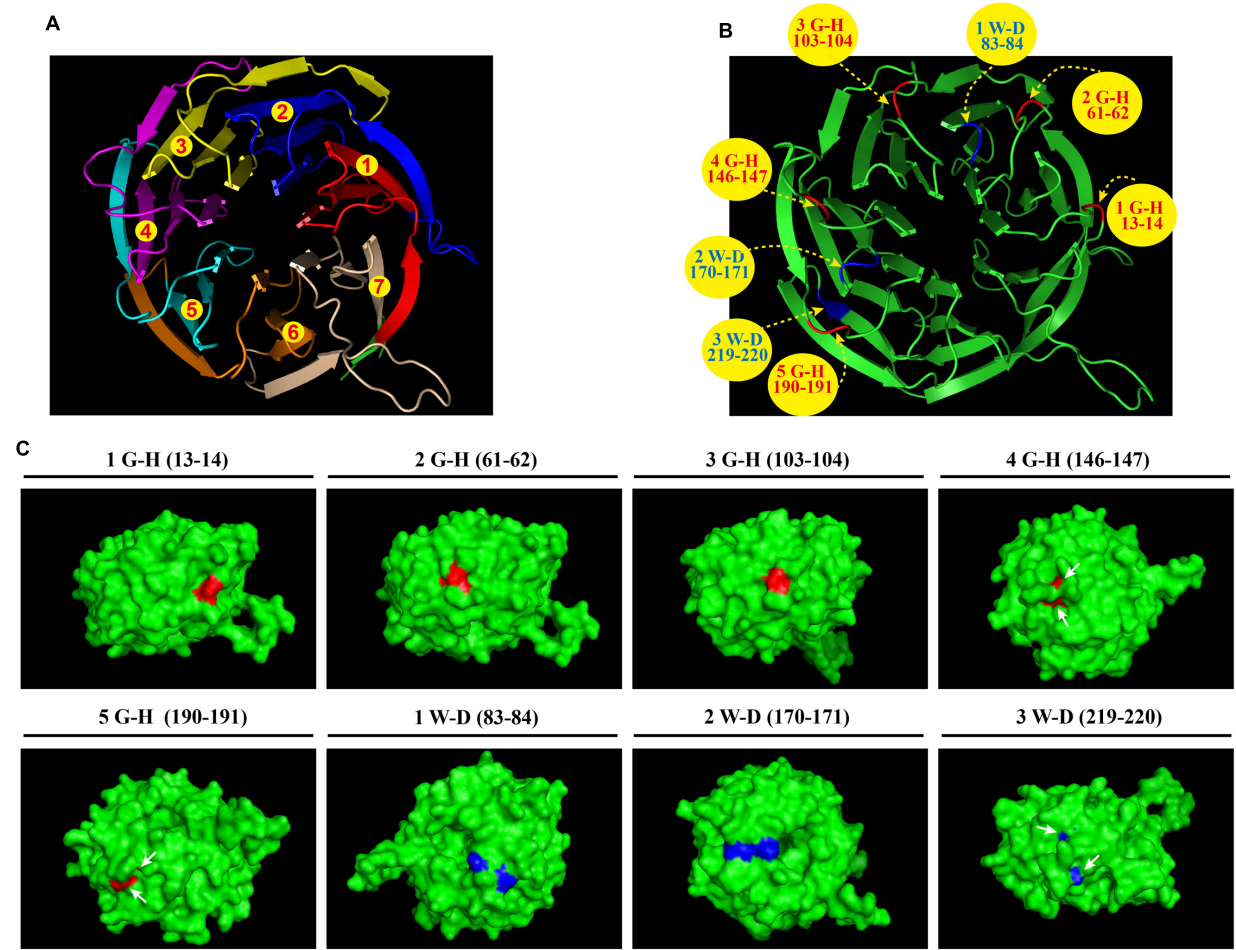
**Three-dimensional CpcB model manipulated and rendered in PyMOL using SWISS-MODEL with the *Oryctolagus cuniculus* 40S ribosomal protein RACK1 as a template.** **(A)** Ribbon diagram of the structure of CpcB showing seven β-propeller blades defined by the seven-numbered WD repeats highlighted in different colors from red to tinted wheat. **(B)** The G–H and W–D residues are colored in red and blue, respectively. **(C)** The G–H residues and W–D residues located on the 3D surface are shown in red and blue, respectively.

Bioinformatics analyses revealed that *A. fumigatus* CpcB contains the conserved G–H and W–D residues that are ubiquitous in WD family proteins. However, the structure and function of the G–H and W–D residues remain to be unclear yet. Therefore, we chose a structurally conserved CpcB homolog, the *Oryctolagus cuniculus* 40S ribosomal protein RACK1, as the template for constructing a 3D homology model using an automated protein homology-modeling SWISS MODEL^1^ Amino acid sequence alignment of CpcB and RACK1 revealed a high level of sequence conservation with an identity of 72.7% (**Supplementary Figure 1**). In addition, information for the secondary structural elements of both proteins indicated that they have a common structure of 28 β-strands, suggesting that RACK1 is a highly conserved homolog of CpcB and may possess a similar crystal structure (**Supplementary Figure 1**). Using the SWISS MODEL, the resulting three-dimensional CpcB model was manipulated and rendered with the biomolecular visualization program PyMOL (Bramucci et al., 2012). As shown in **Figure 1A**, CpcB exhibits a seven-bladed β-propeller structure, with the seven-propeller blades defined by its seven WD sequence repeats. Each propeller blade contains four-stranded antiparallel β sheets, which are composed of the last strand of one blade and the first three strands of the next blade. Although each WD sequence repeat (highlighted in different colors) corresponds to four β strands (highlighted in the same color), it does not correspond exactly to each blade. To better display the position of the conserved G–H and W–D residues, which are common features of WD repeat proteins, we highlighted the G–H residues in red and the W–D residues in blue (**Figure 1B**). Notably, all five G–H dipeptides of CpcB occurred in the loop region connected to the adjacent β strands. In comparison, the W–D residues located at amino acids positions 83–84 and 170–171 were also located in the loop region, excluding the W–D residue at position 219–220 located in the β propeller (**Figure 1B**). Further analysis indicated that the G–H residues at positions 13–14, 61–62, and 103–104 were exposed to the surface of the structure, while those at positions 146–147 and 190–191 were partially buried in the structure (**Figure 1C**). Additionally, in contrast to position 170–171, the W–D residues at positions 83–84 and 219–220 were separated by other residues (**Figure 1C**). Based on the structural characteristics, we found that the G–H and W–D residues at the N-terminus were exposed to the structural surface, while those near the C-terminus were more buried in the structure, suggesting that the G–H and W–D residues at different positions may have different functions and importance.

### The Truncation of WD Repeats in CpcB Affects Normal Hyphal Growth and Conidiation

**FIGURE 2:**
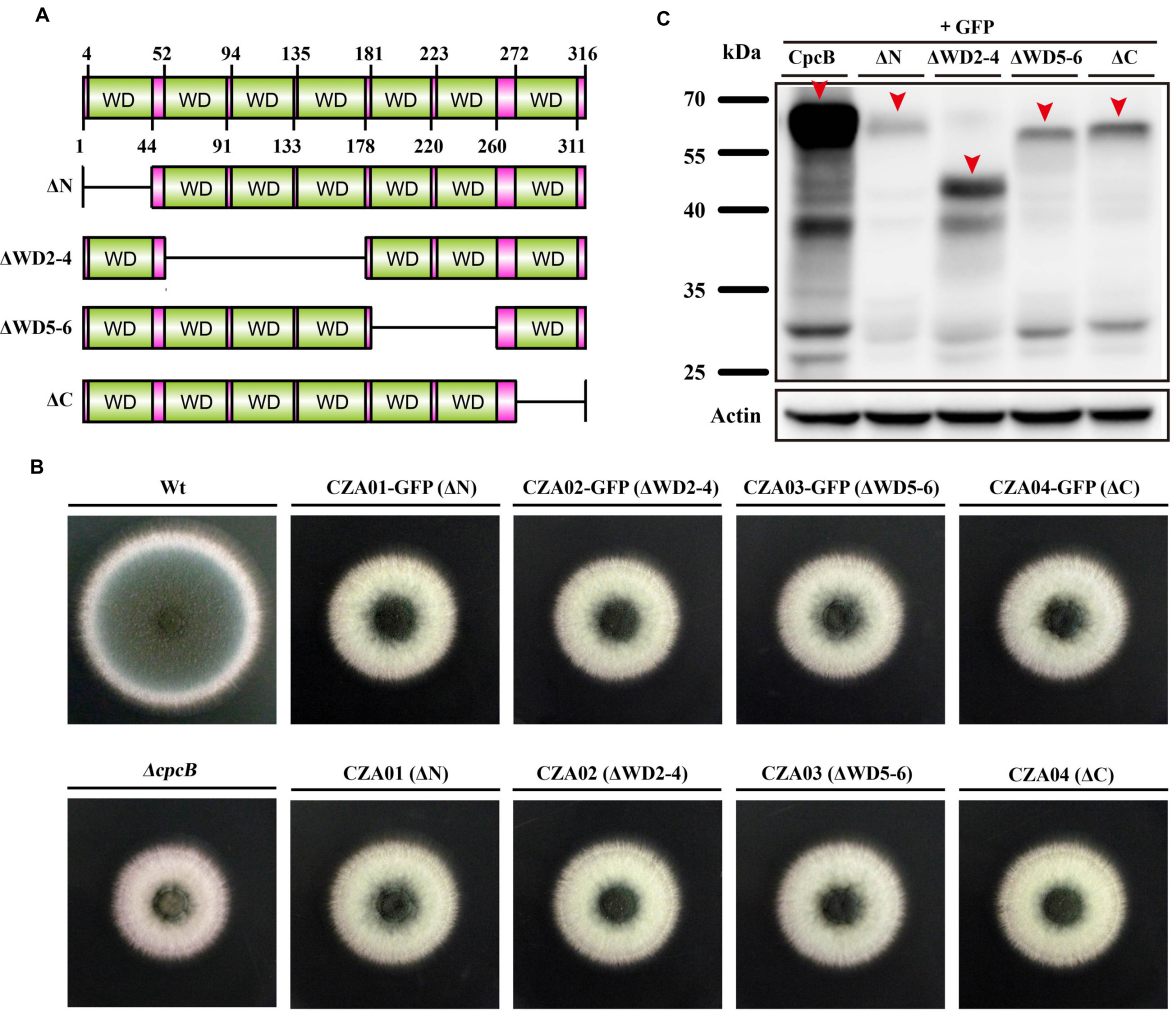
**Deletion of the WD repeats caused severe defects in conidiation and hyphal growth.** **(A)** Schematic representation of the serial WD repeat deletions in CpcB. ΔN: deletion of the region from the second amino acid to WD repeat 1 (2–44 aa); ΔWD2–4: deletion of WD repeat 2–4 (52–178 aa); ΔWD5–6: deletion of WD repeat 5 and 6 (181–260 aa); ΔC: deletion of the region from WD repeat 7 to the terminal amino acid (272–316 aa). **(B)** Colony morphology of the truncated *cpcB* mutants on YAG solid medium at 37°C for 2.5 days. **(C)** Western blotting performed using the anti-GFP antibody showed the predicted molecular masses as indicated.

To identify the function of WD repeats, we performed a serial truncation according to the predicted region of each WD repeat, as shown in **Figure 2A.** We divided seven WD repeats into three regions including N-terminus, middle region and C-terminus. WD repeat 1 and 7 belonged to N-terminus and C-terminus, respectively. Considering the middle region containing WD repeat 2–6 might be large, we made two separate truncations encompassing the WD 2–4 and WD 5–6 domains. Since the GFP-labeled strain can be used to detect not only the expression but also the fluorescence localization of GFP-labeled protein, we constructed related truncated GFP-labeled strains using the same approach used for CpcB-GFP (Cai et al., 2015). For constructing these strains, the CpcB–GFP fusion constructs harboring the corresponding truncated CpcB were transformed into the *ΔcpcB* mutant to generate strains CZA01-GFP (ΔN), CZA02-GFP (ΔWD2-4), CZA03-GFP (ΔWD5-6), and CZA04-GFP (ΔC) with the GFP tag fused at the C-terminus of the truncated CpcB under the control of the native *cpcB* promoter. The phenotype assays revealed that all of the truncated GFP-labeled strains displayed *ΔcpcB*-like defects in radial growth and conidiation on YAG solid medium, which was significantly different from the complemented strain transformed with the full-length *cpcB* gene showing a colony phenotype of WT-like hyphal growth and conidiation (**Figure 2B**). These data suggest that truncated WD repeats in CpcB affect normal hyphal growth and conidiation. Microscopic observation indicated that all the truncated mutants had the GFP fluorescence signal (**Supplementary Figure 2**). Using Western blotting analysis, we previously demonstrated that the CpcB–GFP fusion protein is a polypeptide of approximately 62 kDa (35.0 kDa of CpcB plus 26.9 kDa of GFP). To examine whether the truncated CpcB protein was correctly expressed, we detected the mass of the GFP fusion protein in the GFP-labeled strains by Western blotting analysis with an anti-GFP antibody. Actin was used as a loading control to make sure each lane had the same amount of proteins. As shown in **Figure 2C**, all truncation proteins showed the predicted molecular masses at a position of approximately 57 for CZA01-GFP, 48 for CZA02-GFP, 53 for CZA03-GFP, and 58 kDa for CZA04-GFP, indicating all protein truncation were successful and predicted. Most likely, the weak band near 25 kDa was GFP protein degraded from CpcB–GFP fusion protein. There were same amount of loading protein samples in each lane from actin antibody staining. However, all four truncated protein chimeras were expressed at significantly lower levels as compared to the wild type, which might have contributed to growth and conidiation defects. These findings suggest that the CpcB protein truncation may affect the protein expression. To rule out a possible artificial effect of GFP, we next constructed additional complementation strains referred to as CZA01 (ΔN), CZA02 (ΔWD2-4), CZA03 (ΔWD5-6), and CZA04 (ΔC), using approaches similar to those used to construct the aforementioned CpcB–GFP strains except for the exclusion of the GFP tag. Compared with the *cpcB*-reconstituted strain complemented with full-length *cpcB*, which could completely restore the growth and conidiation defects to wild type levels, all of the CZA01-CZA04 strains still exhibited severe growth and conidiation defects, which was in line with that observed for corresponding GFP-labeled strains (**Figure 2B**).

### The N-Terminal G–H and W–D Residues of CpcB Play More Important Roles than Their C-Terminal Counterparts

**FIGURE 3:**
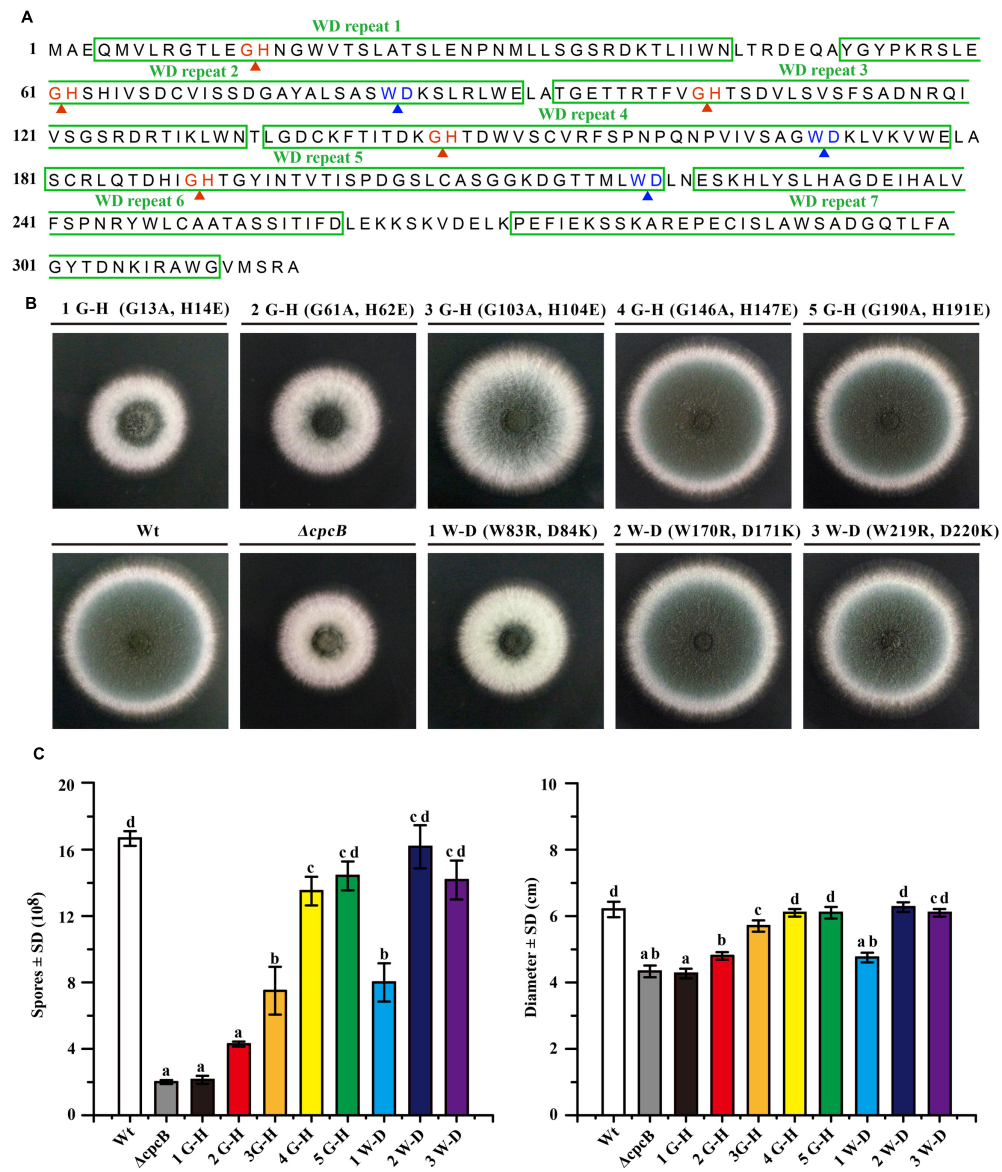
**Comparison of function based on colony phenotypes between the N-terminal G–H and W–D residues and their C-terminal counterparts.** **(A)** Illustration of the amino acid sequences of CpcB protein consisting of the seven WD repeats, including the five G–H and the three W–D residues. **(B)** Colony phenotypes of the parental wild-type (WT) strain and *cpcB* mutants. Equal numbers of conidia (2 × 10^4^) were spotted onto YAG solid medium at 37°C for 2.5 days. **(C)** Quantitative data for the hyphal growth diameters and conidiation after culturing at 37°C for 2.5 days. Values with the same letter are not significantly different, while different letters indicate a significant difference among the tested strains with respect to conidiation and growth (Duncan’s test, *P* < 0.05).

To analyze that loss of function might be due to a conformational change in CpcB caused by the truncation, which was unable to truly reflect the function of the truncated WD repeats, we introduced point mutations at the sites of five G–H and three W–D residues in the WD repeats, which may have differential effects on the function of CpcB based on the analyses of the structural characteristics. To assess the roles of the G–H residues, we generated mutant alleles by substituting Gly-His (G–H) with Ala-Glu (A–E) for all G–H residues in WD repeat 1 to 5 (**Figure 3A**). In these mutants, we individually reintroduced the *cpcB* allele carrying the site-directed mutagenesis driven by the endogenous *cpcB* promoter into the *ΔcpcB* mutant, and referred to the strains as CZA05 (G 13 A, H 14 E), CZA06 (G 61 A, H 62 E), CZA07 (G 103 A, H 104 E), CZA08 (G 146 A, H 147 E), and CZA09 (G 190 A, H 191 E). As shown in **Figure 3B**, colonies of the first and second G–H mutation strains, CZA05 and CZA06, exhibited severe phenotypic defects similar to those observed for the *ΔcpcB* mutant, while the third G–H mutation strain, CZA07, also displayed significantly reduced conidiation and growth defects to some extent. In comparison, there were no detectable defects in the phenotypes of the fourth and fifth G–H mutation strains, CZA08 and CZA09, compared with their control parental WT strain (**Figure 3B**). Using quantitative testing, the production of conidia was found to be less than 50% in the first three G–H mutation strains, while it was more than 80% in the last two G–H mutation strains versus the parental WT strain (**Figure 3C**). Similar to that observed for conidiation, the radial diameter of the hyphal growth was significantly altered in the first three G–H mutation strains compared with the last two mutation strains (**Figure 3C**). In addition, the transcription of *cpcB* in the first three G–H mutation strains, CZA05-CZA07, by RT-PCR confirmed that it was expressed at normal levels (**Supplementary Figure 3**). These results indicate that any of the first three G–H mutations but not the fourth or the fifth G–H mutation has the ability to induce defective conidiation and growth. Therefore, these data indicate that the first three G–H residues at positions 13–14, 61–62, and 103–104 in WD repeat 1, 2, and 3 are required for normal hyphal growth and conidiation, compared with the fourth and fifth G–H residues.

To further test the functional importance of W–D residues in WD repeat 2, 4, and 5 (**Figure 3A**), we substituted Trp-Asp (W–D) with Arg-Lys (R–K) to generate strains CZA10 (W 83 R, D 84 K), CZA11 (W170R, D171K), and CZA12 (W219R, D220K) using the same approach employed to assess the G–H residues. After spotting onto YAG solid medium, the second and the third W–D mutation strain CZA11 and CZA12 displayed WT-like growth and conidiation, probably CZA12 had a modest effect (if any), suggesting that they might not contribute to the function of CpcB (**Figure 3B**). In contrast, the first W–D residue mutation strain, CZA10, showed remarkably defective growth and conidiation, similar to that of the mutant carrying the full-length *cpcB* deletion. These results suggest that the first W–D residue at amino acid position 83–84 play a more important role in hyphal growth and conidiation than the second and the third W–D residues. The quantification data indicated that CZA10 produced 48% of the conidia and 77% of the colony diameter of the parental WT strain; the third W–D residue mutation strain, CZA12, produced approximately 85% of the conidia and 98% of the colony diameter of its parental WT strain (**Figure 3C**). These results further suggest that the first W–D residue at position 83–84 in WD repeat 2 have a more significant effect on conidiation and hyphal growth than others. Taken together, on the basis of the structural characteristics of exposure surface of CpcB combined with phenotypes in site-mutations, the N-terminal G–H and W–D residues of CpcB are required in hyphal growth and conidiation. However, it could not exclude the possibility that site-mutations may affect the colony growth through the decreased protein expressions which are similar to that of truncated CpcB.

### Defects in Full-Length CpcB or N-Terminal G–H and W–D Residues Result in Multidrug Antifungal Resistance

**FIGURE 4:**
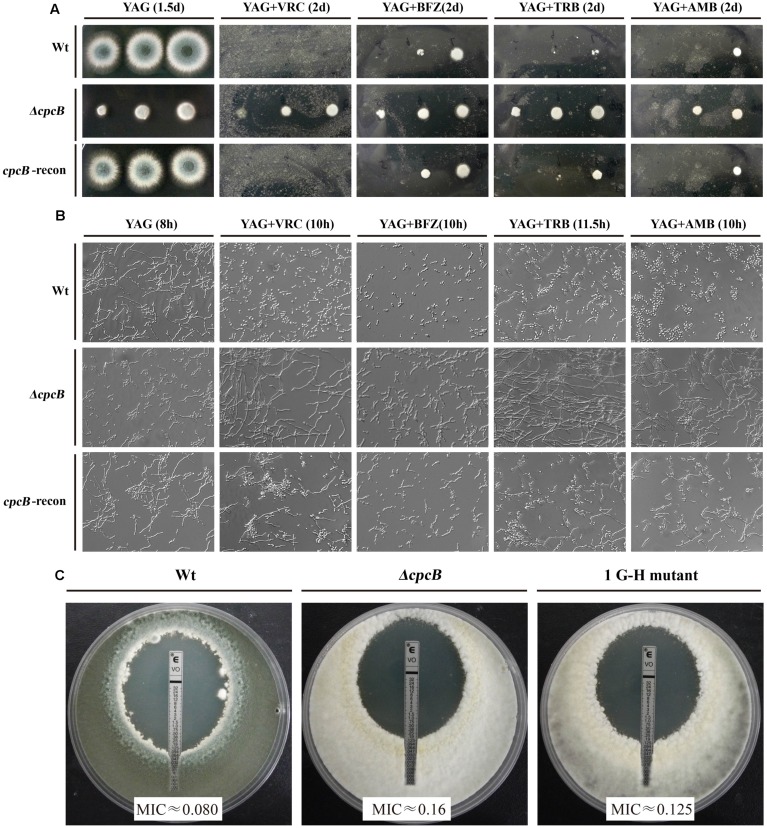
***cpcB* mutants caused multidrug resistance based on the results of the drug susceptibility assays.** **(A)** Equal numbers of conidia (2 × 10^4^) were inoculated onto YAG solid medium without drug at 37°C for 1.5 days or with drug at 37°C for 2 days. The following antifungal drugs were used: 0.35 μg/ml voriconazole (VRC), 1.5 μg/ml bifonazole (BFZ), 0.45 μg/ml terbinafine (TRB), and 10 μg/ml amphotericin B (AMB), respectively. **(B)** Equal numbers of conidia were cultured at 37°C for the indicated times in YG liquid culture without drug addition or with 0.18 μg/ml voriconazole (VRC), 0.8 μg/ml bifonazole (BFZ), 0.25 μg/ml terbinafine (TRB), or 10 μg/ml amphotericin B (AMB), respectively. **(C)** MIC *E*-test strips impregnated with a gradient of VRC were placed onto a YUU agar plates containing a lawn of conidia cultured for 48 h at 37°C before observation.

Several lines of evidence support the notion that G protein-mediated signaling contributes to the antifungal drug response (da Silva Ferreira et al., 2006; Lafayette et al., 2010). To verify whether Gβ-like CpcB participates in the antifungal drug susceptibility, we performed a transcriptome analysis to identify a possible link between CpcB and drug susceptibility resulting from the differential expression of genes related to the drug response. As shown in **Supplementary Table 2**, ergosterol biosynthesis-related genes, the putative *erg5*, *erg10*, and *erg26* orthologs and the ABC multidrug transporter gene-*mdr1* in *A. fumigatus*, were significantly differentially expressed in the *ΔcpcB* mutant compared with the control parental WT strain. To verify a potential link between these changes in expression and drug responses, we performed drug susceptibility testing using three classes of antifungal compounds, including triazoles (voriconazole and bifonazole), the non-azole ergosterol inhibitors allylamines (terbinafine) and polyenes (amphotericin B). Conidia from the parental WT, *ΔcpcB* mutant, and *cpcB*-reconstituted strains were serially diluted and spotted onto YAG solid medium with or without drug addition. Although it displayed small colonies on YAG solid medium, the *ΔcpcB* mutant exhibited a measurable increase in drug resistance compared with the parental WT and *cpcB*-reconstituted strains under all of the tested antifungal drug conditions, especially on medium containing voriconazole, in which all three droplets of the *ΔcpcB* mutant conidia produced visible colonies (**Figure 4A**). However, even for the maximum droplet of conidia, the parental WT and the *cpcB*-reconstituted strains failed to show any detectable colonies under the same condition. These results suggest that the *ΔcpcB* mutant has the enhanced resistance ability against voriconazole, bifonazole, terbinafine, and amphotericin B. To further examine whether antifungal drug resistance could occur under the liquid culture condition, we examined the growth of the *ΔcpcB* mutant in YG liquid medium in the presence or absence of the above-mentioned antifungal drugs. Consistent with the previous findings, the *ΔcpcB* mutant exhibited a phenotype with shortened hyphae compared with the parental WT and *cpcB*-reconstituted strains in liquid YG medium without any antifungal drug. In comparison, the *ΔcpcB* mutant exhibited more robust growth than the parental WT and *cpcB*-reconstituted strains in the presence of all of the tested drug culture conditions (**Figure 4B**), suggesting that the deletion of *cpcB* results in increased drug resistance under the liquid drug culture conditions. To further quantify the resistance ability in these mutants, the minimal inhibitory concentration (MIC) assay was used. All data for the growth optical density (O.D) percentage ratio with drug versus without drug in *cpcB* mutants at three tested drug concentrations (except for BFZ at the concentration of 0.16 μg/ml, and AMB at 88 μg/ml) were significantly higher than that in parental WT strain, suggesting the *cpcB* mutants enhanced resistance against voriconazole, bifonazole, and amphotericin B (**Table 2**). Further analysis by MIC *E*-test strips showed that the value of VRC in *ΔcpcB* mutant was higher than that in its parental WT strain based on observed cleared areas that represent fungal growth inhibition by VRC (**Figure 4C**). Taken together, these results suggest that CpcB is responsible for maintaining normal antifungal drug susceptibilities.

**Table 2:** The drug susceptibility analysis in a liquid 96-well culture plate.

Drug concentration (μg/ml)		O.D_drug_/O.D_no drug_ × 100% ± SD^a^		
		
		Wt	Δ*cpcB*	1 G–H mutant
VRC	0	100	100	100
	0.045	81.91 ± 0.24	91.12 ± 0.49^∗^	87.66 ± 0.22^∗^
	0.09	77.86 ± 0.20	90.42 ± 0.23^∗^	85.57 ± 0.40^∗^
	0.18	75.96 ± 0.54	85.04 ± 0.78^∗^	81.92 ± 0.64^∗^
BFZ	0	100	100	100
	0.16	53.72 ± 0.48	47.28 ± 0.40^∗^	53.88 ± 0.32^∗^
	0.32	31.38 ± 0.05	38.52 ± 0.11^∗^	38.41 ± 0.22^∗^
	0.64	32.82 ± 0.42	37.56 ± 0.22^∗^	36.79 ± 0.15^∗^
TRB	0	100	100	100
	1	49.80 ± 0.82	51.54 ± 0.29^∗^	62.11 ± 0.70^∗^
	2	34.52 ± 0.98	45.07 ± 0.36^∗^	53.64 ± 0.58^∗^
	4	30.38 ± 0.30	33.68 ± 0.03^∗^	34.87 ± 0.17^∗^
AMB	0	100	100	100
	88	40.65 ± 0.06	40.85 ± 0.05	41.13 ± 0.06
	176	36.26 ± 0.02	41.26 ± 0.06^∗^	41.48 ± 0.17^∗^
	352	40.24 ± 0.10	45.41 ± 0.04^∗^	46.15 ± 0.36^∗^


*^a^Data are shown as means ± SD for three independent replicates. Asterisk represents the significant difference from the parental wild-type (Wt) strain under the same drug concentration. (Duncan’s multiple range tests, *P* < 0.05).*

**FIGURE 5:**
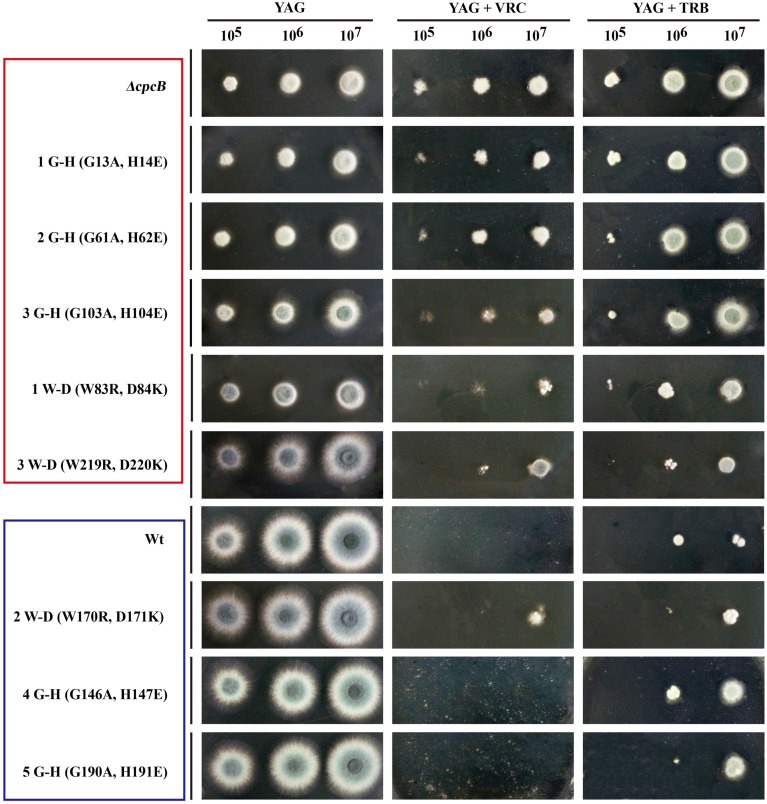
**Phenotypic characterizations of G–H and W–D mutation strains grown in the presence of the tested drugs.** Equal numbers of conidia (2 × 10^4^) were inoculated onto YAG solid medium at 37°C for 1.5 days in the absence of drug or in the presence of 0.35 μg/ml voriconazole (VRC) and 0.45 μg/ml terbinafine (TRB) at 37°C for 2 days.

Because the aforementioned data indicated that mutations in G–H and W–D residues correlated with the function of CpcB in growth and conidiation, we wondered whether G–H and W–D residues are involved in CpcB-mediated drug responses. Thus, we performed drug susceptibility assays. As shown in **Figure 5**, *cpcB* mutants including the first three G–H residues and the first one W–D residue mutation strains (labeled in the red frame) showed comparable drug resistance phenotypes to the *ΔcpcB* mutant in response to the selected drug conditions, despite exhibiting relatively slow hyphal growth on YAG solid medium without drug addition. In line with the observed resistance phenotype, the first G–H mutation strain showed an increase of MIC for VRC with *E*-test strips than the parental strain did (**Figure 4C**). These results suggest that the first three G–H and the first one W–D dipeptides play a crucial role in drug responses, followed by the third W–D residue. The mutants labeled in the blue frame exhibited WT-like colony phenotypes regardless of the presence or absence of drugs. These results suggest that the second W–D and fourth and fifth G–H residues might not confer CpcB-mediated drug susceptibility. Therefore, we conclude that the N-terminal G–H and W–D residues that are required for growth and conidiation are also necessary for maintaining normal antifungal drug susceptibility.

### *cpcB* Mutants Cause the Decrease in R6G Accumulation as Well as the Altered Ergosterol Component

**FIGURE 6:**
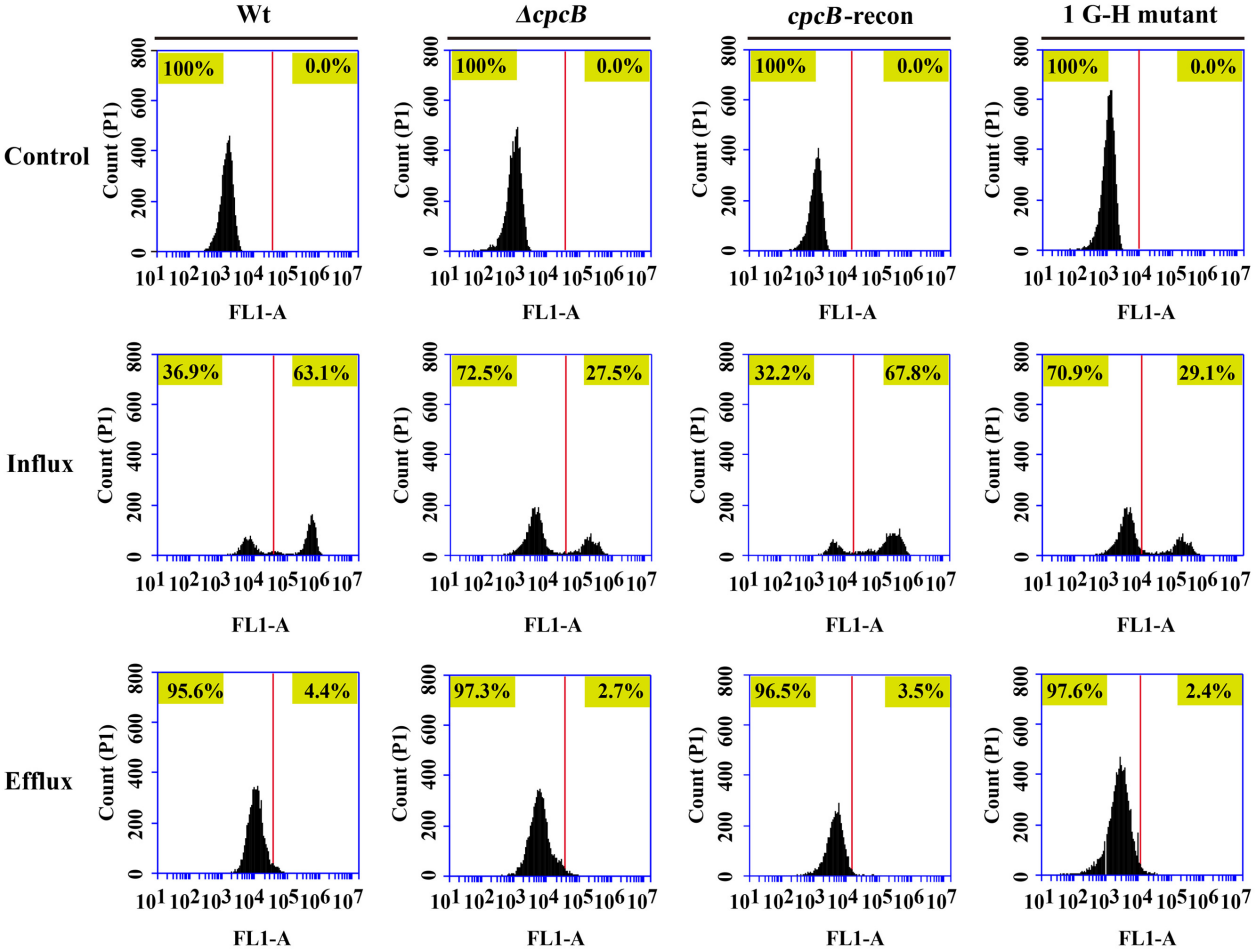
**Comparative analyses of the *cpcB* mutants and parental strain by flow cytometry.** The *cpcB* mutants showed a decrease in R6G accumulation. FL1-A on the X-axis represents the relative fluorescence intensity value.

To identify the possible causes of the drug resistance, we examined the intracellular accumulation of antifungal drugs, which is one of the main mechanisms of drug susceptibility across fungal pathogens (Sanglard and Odds, 2002; Chamilos and Kontoyiannis, 2005; Prasad and Kapoor, 2005). The accumulation of drug in the cell results from the balance between import into and efflux out of the cell. Therefore, we investigated both the influx and efflux of the drug using a fluorescent dye-rhodamine 6G (R6G), which is a drug molecule-mimicking substrate and is extruded by transporters from cells in an energy-dependent manner (Maesaki et al., 1999; Mailloux et al., 2014). Among the *cpcB* mutants that showed a correlation with drug susceptibility, we chose the first G–H mutation strain as a representative mutant to analyze the drug susceptibility. The autofluorescent signal generated by unlabelled cells without R6G was used as a negative control for each tested sample. As shown in **Figure 6**, the ratio of the intracellular R6G fluorescence intensity to the total intracellular fluorescence intensity was 27.5% in the *ΔcpcB* mutant and 29.1% in the first G–H mutation strain CZA05; however, it reached 63.1% in the parental WT strain and 67.8% in the *cpcB*-reconstituted strain. Consistent with these ratios, the relative mean fluorescence intensity of R6G accumulation was 51.6 ± 3.1 arbitrary units for the WT strain, which was more than double the amount determined for the *ΔcpcB* mutant (23.7 ± 1.7 arbitrary units) or the first G–H mutation strain CZA05 (22.6 ± 2.0 arbitrary units), suggesting that significantly reduced R6G uptake/influx occurred in both *cpcB* mutants compared with the parental WT and *cpcB*-reconstituted strains based on three independent experiments. After glucose-induced R6G efflux, the ratio was comparatively lower in *cpcB* mutants compared to its parental WT and *cpcB*-reconstituted strains. Therefore, we concluded that both the *ΔcpcB* mutant and the first G–H residue mutant CZA05 had a decrease in R6G accumulation, which might account for the enhanced drug resistance.

In addition to the reduced intracellular accumulation of antifungal drugs, modification of the ergosterol biosynthetic pathway is another possible explanation for the observed antifungal resistance (Prasad et al., 1995; Sanglard et al., 1995; White, 1997; Sanglard, 2002; Chamilos and Kontoyiannis, 2005). Thus, we compared the levels of ergosterol in parental WT strain, *ΔcpcB* mutant, *cpcB*-reconstituted strain, and the selected G–H mutation strain CZA05 by high-performance liquid chromatography (HPLC) analysis. Total ergosterol in the *A. fumigatus* strains was extracted after 18 h of growth in liquid MM using a previously described method (Alcazar-Fuoli et al., 2008). We observed a major increase at approximately 11.25 min of retention time, indicating an increase in ergosterol content in the *cpcB* mutants compared with the parental WT and *cpcB*-reconstituted strains based on repeated tests (**Supplementary Figure 4A**). According to the quantitative analysis of ergosterol content, the *ΔcpcB* mutant displayed a significantly different change for this increasing peak compared with both the control parental WT and the *cpcB*-reconstituted strains; however, the G–H mutation strain did not exhibit any apparent changes compared with other strains (**Supplementary Figure 4B**). Further analysis showed that the ratio of the two main peaks in **Supplementary Figure 4A** was significantly different in both *cpcB* mutants compared with the parental WT and *cpcB*-reconstituted strains, respectively (Duncan’s test, *P* < 0.001; **Supplementary Figure 4C**). Therefore, we conclude that both the strains carrying the *cpcB* deletion and the first G–H mutation have altered ergosterol contents, which might correlate with the increase in drug resistance observed in the related *cpcB* mutants.

### Mutation of the First G–H Residue Results in Significantly Attenuated Virulence in an Immunosuppressed Mouse Model

**FIGURE 7:**
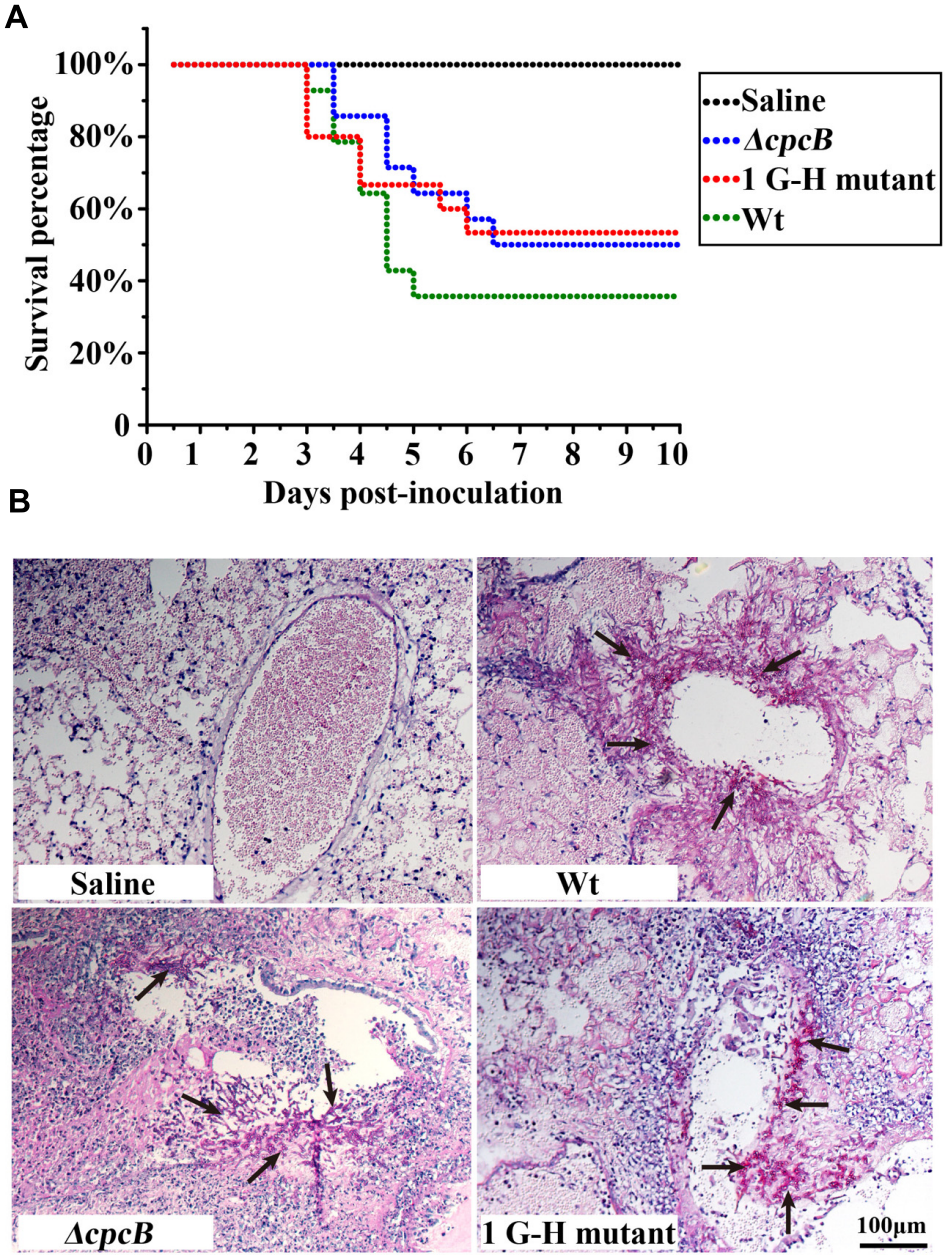
**Virulence testing of the strain carrying the first G–H mutation in an immunosuppressed mouse model.** **(A)** The strain carrying the first G–H mutation showed attenuated virulence, similar to the *ΔcpcB* mutant based on data for the survival rate. **(B)** Histopathological analyses of lung tissue from mice sacrificed at day 5 post-inoculation were conducted using Periodic acid-Schiff (PAS) staining. Compared with the WT strain, the lungs from mice inoculated with the strain containing the G–H mutation exhibited clearly reduced invasive fungal growth compatible to that observed for the *ΔcpcB* mutant.

Because the mutants at the first three G–H residues in WD repeat 1–3 and the W–D residue in WD repeat 2 showed not only defective hyphal growth and conidiation but also enhanced drug resistance, similarly to the *cpcB* full-length deletion mutant, we wondered whether they would show differences in virulence, which was significantly decreased in the *ΔcpcB* mutant compared to the parental WT strain and the *cpcB*-reconstituted strain (Cai et al., 2015). Since the mice infected by *cpcB*-reconstituted strain had a similar survival curve to that of the parental WT (Cai et al., 2015), we established the mice virulence test only including *cpcB* deletion mutant, 1G–H mutant and the parental WT strain using highly immunosuppressed mice. According to the same approach described previously (Cai et al., 2015), conidia of parental WT, *ΔcpcB* mutant and G–H mutant strains were inoculated into the immunocompromised mice via endotracheal intubation, and the mice in the saline group received 0.3 ml of saline alone. Based on the survival curve (**Figure 7A**), more than 50% of the mice inoculated with the G–H mutant but only 35% of those inoculated with the control parental WT strain survived to the experimental end-point. Moreover, Kaplan–Meier log-rank analysis showed there was a significant difference between these two groups (*P*-value = 0.002), further suggesting that the survival rate of mice infected with the G–H mutant was significantly higher than that of the mice infected with the control parental WT strain. In comparison, mice infected with the G–H mutant had a similar survival rate to the mice infected with the *ΔcpcB* mutant (P value = 0.633, log-rank test). To further dissect the difference between the G–H mutant and the parental WT strain, we conducted histopathological analyses using the lungs of mice sacrificed at day 5 post-inoculation. Periodic acid-Schiff (PAS) staining revealed that the lungs of mice inoculated with the parental WT strain had aggressive fungal growth that spread into the lung parenchyma compared with the marginal growth in the lungs of mice inoculated with the G–H mutant, suggesting that the G–H mutant was efficiently controlled by the host immune system (**Figure 7B**). These data suggest that the first G–H residue of CpcB play an important role in the virulence of *A. fumigatus*.

## Discussion

Invasive fungal infections pose a serious health risk to immunocompromised individuals. However, specific antifungal drugs with few side effects remain limited due to the limited fungal-specific drug targets because of the close evolutionary relationships of these eukaryotic pathogens with their hosts (Hill et al., 2013). Therefore, it is urgently necessary to evaluate specific peptide regions in drug target proteins and virulence-related proteins to target regions that can kill or inhibit pathogen viability while causing fewer side effects in the host. To achieve this goal, molecular characterization is widely used to identify key motifs of virulence-related proteins. Despite its important functions in hyphal growth, conidiation, and virulence, the molecular characterization of CpcB has not been yet conducted; thus, the relative functional motifs and crucial peptides in this protein remain unknown. In the present study, we demonstrate that the G–H residues in WD repeat 1, 2, 3, and the W–D dipeptide in WD repeat 2 are required not only to control normal hyphal growth and conidiation but also affect antifungal drug susceptibility, suggesting that N-terminal G–H and W–D peptides play crucial roles in the function of CpcB, especially in virulence. Our findings reveal the function of G–H and W–D residues of CpcB, which may have a broad spectrum of importance for conserved Gβ-like homologs and WD repeat proteins in fungal pathogens. However, further biochemical evidence and the crystal structure of CpcB will provide a better understanding of the importance of G–H and W–D residues.

The heterotrimeric G protein complex comprises Gα, Gβ, and Gγ subunits and transduces signals to effectors in all eukaryotes (Shukla et al., 2014; Dohlman, 2015; Walther and Ferguson, 2015). The circular β-bladed propeller structure of the Gβ protein confers interactions of Gβγ with a variety of proteins to perform diverse functions (Chen et al., 2004). In addition, the Gβ-like/RACK homolog Gib2 functions as a scaffold protein and interconnects diverse cellular processes in *Cryptococcus neoformans* (Ero et al., 2015). Furthermore, crystal structure of Gib2 showed its versatile functions as a ribosome-bound scaffold to recruit various proteins to ribosomes functioning in ribosomal biogenesis and protein translation (Ero et al., 2015). Gβ-like CpcB that is abundantly distributed in the cytoplasm and possesses a similar crystal structure of Gib2 may interconnect ribosome proteins in a similar manner. Based on the 3D homology model of CpcB, functional G–H and W–D residues are mainly located in the loop region and exposed to the surface of the structure. Therefore, we deduced that the exposed G–H and W–D residues are accessible for protein–protein interactions and that mutation of these sites might change the conformation of the loop region, preventing the mutual recognition, interaction and thus signal transduction.

Moreover, based on the 3D model hypothesis for CpcB’s function, we firstly demonstrated the functional importance of WD repeats by truncation experiment. However, we found four truncated CpcB mutants could cause the significantly lower protein expression, which resulted in growth defects compared to that of their parental WT strain. Although there were some of degraded GFP fragment proteins in mutants, compared to the parental WT strain, all generated truncated CpcB were expressed at significantly lower levels as compared to the parental WT strain, therefore, it remains to be unsure whether the correlations can be established between the defective phenotypes and WD repeats truncations due to the lower CpcB protein level caused by truncation. Therefore, the defects of hyphal growth and conidiation might be a consequence of either protein truncation or lower protein expression or both in truncated mutants. It also indicates limitations for interpreting truncation approaches for studying the function of CpcB. On the other hand, there was another possibility that *cpcB* mutants grew very slowly and sick compared to its parental strain, and possibly it was due to the different development stages with different protein levels between the *cpcB* mutants and the parental WT strain. Therefore, we switched to make site-direct mutants. Our experimental data showed that the mutants carrying modifications in the N-terminal G–H and W–D residues had similar defects in hyphal growth and conidiation to that carrying the full-length *cpcB* deletion. Because the protein levels of CpcB were not estimated in the point mutants, it is unsure whether the sick phenotype also resulted from decreased protein expression similar to that in truncated mutants. Further study using fusion tag will address this puzzle. In addition, the drug susceptibility testing exhibited multidrug antifungal resistance in *cpcB* mutants, reflected by enhanced resistance to kinds of antifungal drugs belonging to the triazole, polyene, and allylamine drug classes, which are widely used for the treatment of invasive aspergillosis (Alcazar-Fuoli and Mellado, 2012). Among the triazole antifungals, bifonazole, and voriconazole inhibit ergosterol synthesis through P-450 cytochrome-mediated lanosterol demethylation (the drug target is Cyp51/Erg11), leading to toxic sterol accumulation and cell death (Ghannoum and Rice, 1999; Martel et al., 2010; Alcazar-Fuoli et al., 2011). The allylamine terbinafine inhibits the enzyme squalene epoxidase (Erg1) at the early stage of ergosterol biosynthesis (Liu et al., 2004; Ruckenstuhl et al., 2007). The polyene amphotericin B functions via channel-mediated membrane permeabilization, leading to fungal cell death (Gray et al., 2012). In addition, the resistance phenotype to amphotericin B associated with mutations in *erg3* has been extensively investigated in yeasts (Kelly et al., 1997; Morio et al., 2012; Zavrel and White, 2015). Based on the analysis of drug targets, we conclude that all three major groups of antifungal agents owe their antifungal activities to the inhibition of ergosterol synthesis or direct interaction with ergosterol, which is the major component of the fungal cell membrane. Therefore, we speculate that CpcB is involved in various steps of the regulation of the ergosterol biosynthetic pathway. Ergosterol, the main component of fungal membranes, is essential for developmental growth and is well known as the target of many clinically used antifungals. Based on the multidrug resistance phenotype associated with *cpcB* mutation, we hypothesize that the ergosterol synthesis in the *cpcB* mutants was influenced significantly. To verify our hypothesis, we examined the ergosterol content by HPLC analysis, and the *cpcB* mutants displayed significant changes in ergosterol peaks compared with the parental WT strain. Since they had enhanced ergosterol contents and Erg11 (14-α sterol demethylase) is a target of azole drugs. Most likely, the expression of Erg11 could be increased in *cpcB* mutant. However, as shown in **Figure 4**, the *cpcB* mutants could result in multidrug antifungal resistance not specific for azole antifungals. Therefore, it suggests that probably there are multiple resistance mechanisms. On the other hand, our flow cytometry analysis data indicated a reduced uptake of the drug molecule-mimicking substrate-R6G by the *cpcB* mutants compared with the control parental strain, suggesting that the deletion of *cpcB* might result in structural or component changes in plasma membrane. Furthermore, the transcriptome data showed that CpcB affects the transcription of a large number of genes, including undefined genes with various biological functions such as metabolic processes, oxidation reduction processes, transmembrane transport, and response to stress by the analyses of Gene Ontology (GO) classification (**Supplementary Table 3**; Priebe et al., 2011). The raw Illumina sequencing data were deposited in SRA^2^ at NCBI with accession numbers SRR3098053 and SRR3098040. These findings suggest that in addition to being a member of the G protein signaling system in *A. fumigatus*, CpcB may play multiple unexplored roles during the antifungal drug response.

A few studies have suggested that G protein signaling pathways are associated with antifungal drug susceptibility. For example, the cAMP-PKA signaling pathway mediates azole drug susceptibility in *Candida albicans*, *S. cerevisiae*, and *A. fumigatus* (Kontoyiannis and Rupp, 2000; Jain et al., 2003; da Silva Ferreira et al., 2006). Moreover, Lafayette et al. (2010) demonstrated that PKC signaling regulates drug resistance via the circuitry consisting of Mkc1, Calcineurin, and Hsp90 in *C. albicans*. Based on our data for Gβ-like CpcB-mediated drug susceptibility together with the results of others, we conclude that the G protein complex plays important roles in maintaining antifungal drug susceptibility.

## Author Contributions

Conception and design of the investigation and work: ZC, LL, and HS. Completion of the experiments: ZC, YC, CZ, and RF. Evaluation and analysis of the results: ZC, YC, and LL. Manuscript writing: ZC and LL. Final approval of manuscript: ZC, YC, CZ, RF, HS, and LL.

## Conflict of Interest Statement

The authors declare that the research was conducted in the absence of any commercial or financial relationships that could be construed as a potential conflict of interest.

## Footnotes

1 http://swissmodel.expasy.org/.

2 http://www.ncbi.nlm.nih.gov/sra
