# Supplementary material for: Molecular Characterization of Gβ-Like Protein CpcB Involved in Antifungal Drug Susceptibility and Virulence in A. fumigatus
Source: Front Microbiol. 2016 Feb 9;7:106. doi: 10.3389/fmicb.2016.00106 (PMC4746314; doi:10.3389/fmicb.2016.00106)
Supplement: Supplementary file 1 [file Presentation_1.PDF]

Supplementary Material

Molecular characterization of Gβ-like protein CpcB involved in antifungal drug susceptibility and virulence in *A. fumigatus*

Zhendong Cai[1], Yanfei Chai[1], Caiyun Zhang[2], Ruoyun Feng[1], Hong Sang[2] and Ling Lu[1,*]

[1] Jiangsu Key Laboratory for Microbes and Functional Genomics, Jiangsu Engineering and Technology Research Center for Microbiology; College of Life Sciences, Nanjing Normal University, Nanjing, 210023, China
[2] Department of Dermatology, Jinling Hospital, School of Medicine, Nanjing University, Nanjing, 210023, China

*Corresponding author: Ling Lu
College of Life Sciences
Nanjing Normal University
No.1 Wen Yuan Rd, Qi Xia Qu, Nanjing, China
Phone/Fax: +86-025-85891791
E-mail: linglu@njnu.edu.cn

1 Supplementary Figures and Tables

1.1 Supplementary Figures

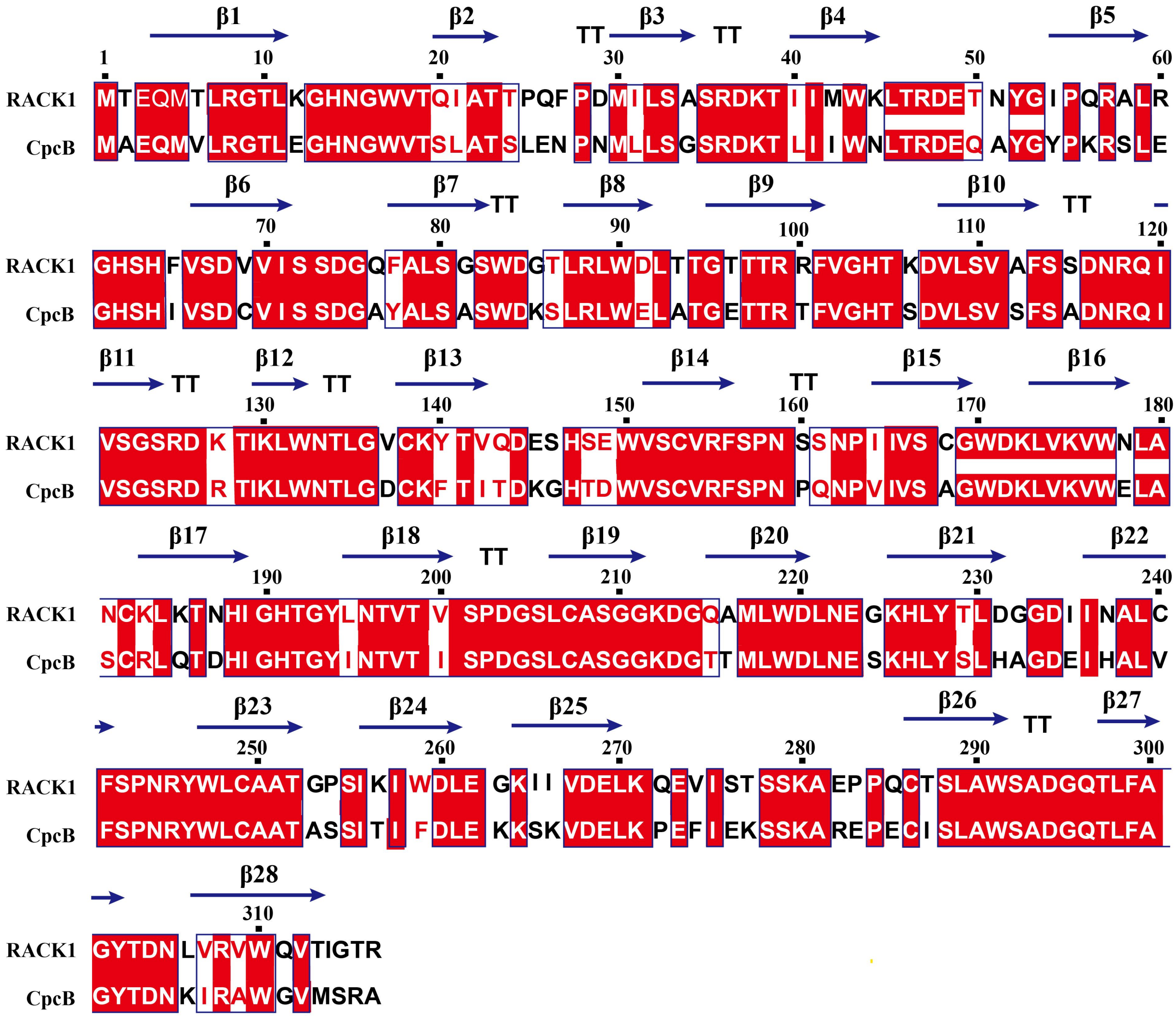

Supplementary Figure 1. Structure-based sequence alignment of CpcB and RACK1. White letters in a red background show the strict identity; red letters in a white background show the similarity; and blue box shows a similarity across groups. The secondary structure elements of both proteins are labeled β (β-strand) and TT (turn). The alignment was performed with ClustalX2.1 (Thompson et al., 1997), manually modified based on the structures of CpcB and RACK, and represented with ESPript (Gouet et al., 1999).

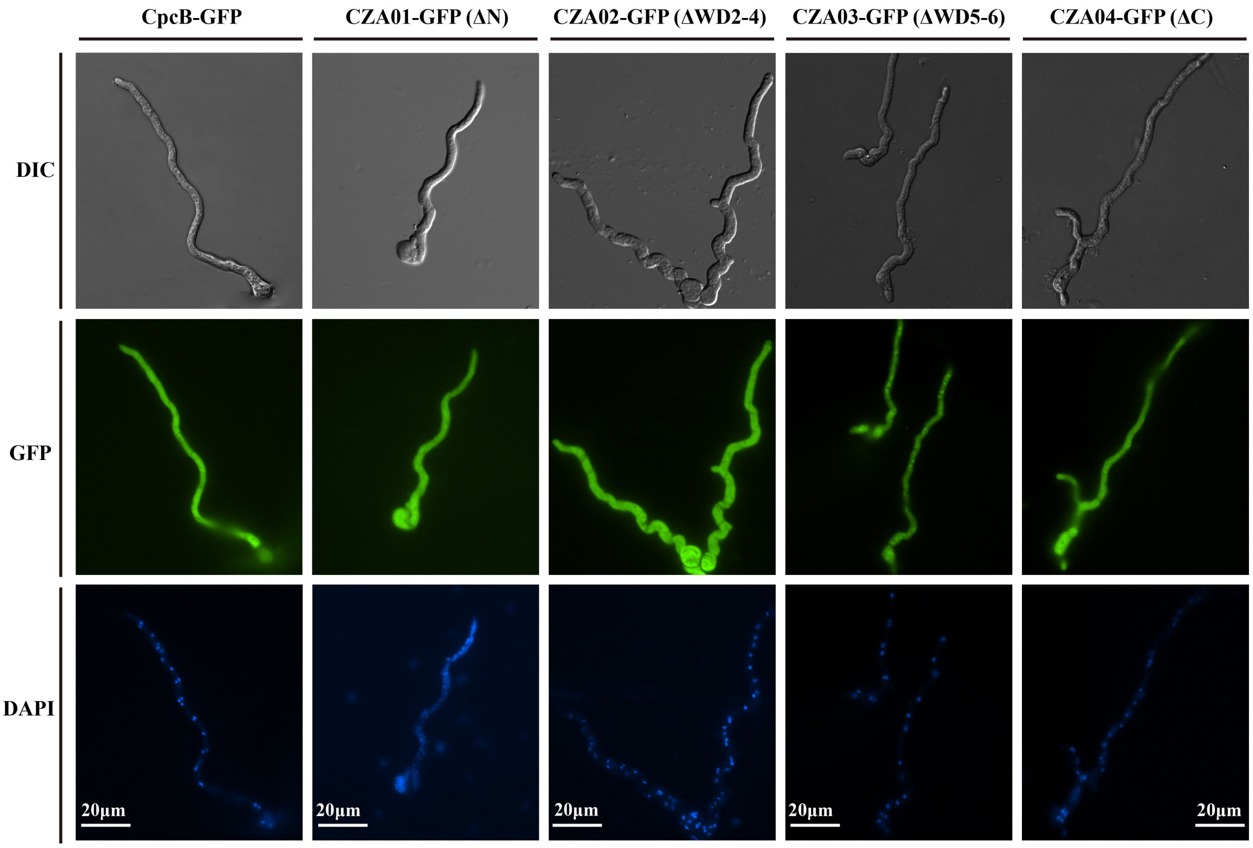

Supplementary Figure 2. The indicated GFP labelled strains showed strong GFP fluorescence signals. DIC (differential interference contrast) was used to show the shapes of hyphae. DAPI was used to visualize nuclei. GFP fluorescence showed the location of the indicated CpcB. Scale bar = 20 μm.

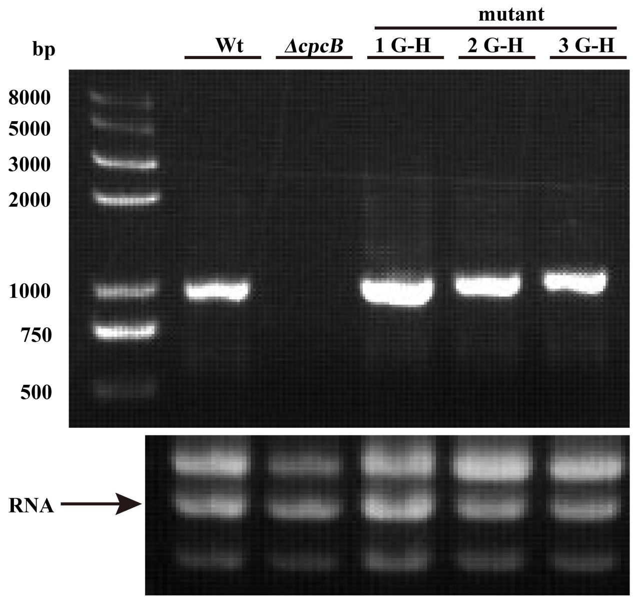

Supplementary Figure 3. RT-PCR analysis of *cpcB* transcription. As a negative control for the *ΔcpcB* mutant, *cpcB* was expressed at an equivalently comparable level

of Wt in the first three G-H mutation strains.

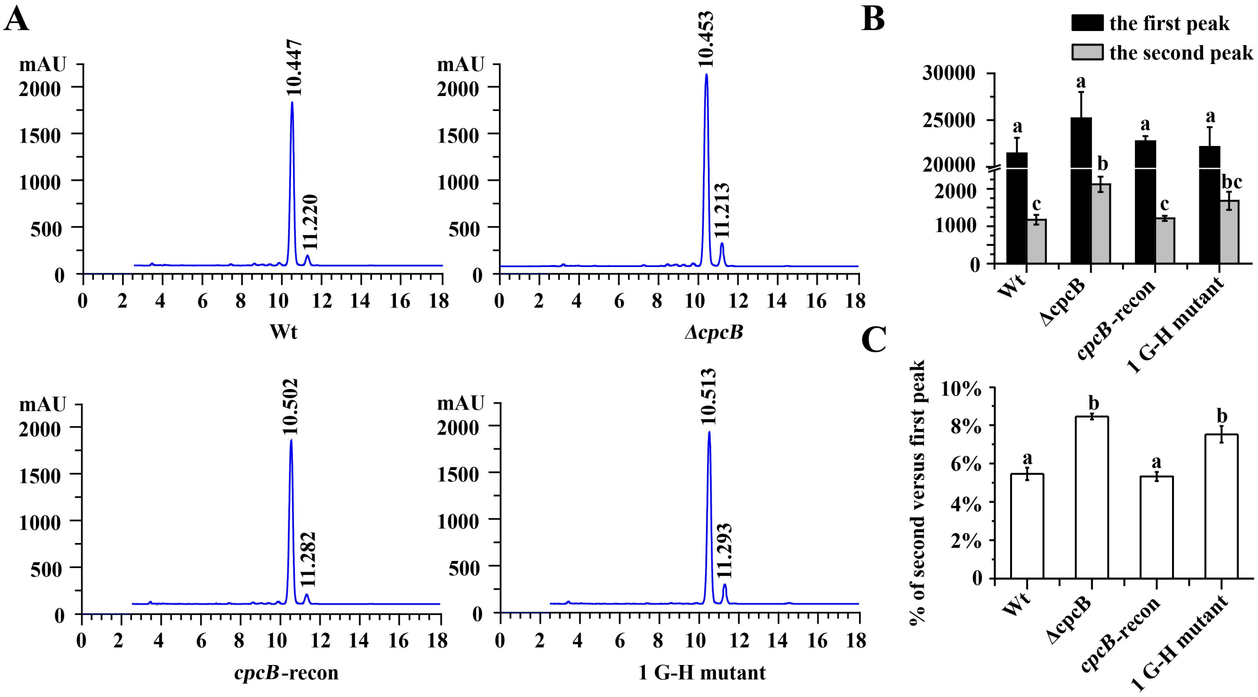

Supplementary Figure 4. *cpcB* mutants resulted in the altered ergosterol contents compared to the parental wild-type and *cpcB*-reconstituted strains based on representative HPLC analysis. (**A**) Reversed-phase HPLC analysis was performed using total ergosterol at a wavelength of 282 nm. Units are given in milli absorption units (mAu). (**B**) Ergosterol contents of the first and second peaks at approximately 10.5 and 11.25 min were respectively compared among the indicated strains (Duncan's test, P≤ 0.05). (**C**) The ratio of the two peaks in the *cpcB* mutants showed a significant difference from the parental wild-type and *cpcB*-reconstituted strains (Duncan's test, P≤ 0.001). In (**B**) and (**C**), different lowercase letters on the bars indicated significant differences in values, while the same letters showed no significant differences.

1.2 Supplementary Tables

Supplementary Table 1. Primers used in this study

Primer/Purpose	Sequence (5' - 3' direction)	Reference
**Plasmid pCA01: CZ-P01/CZ-P07, GFP-F/GFP-R, CZ-P05/CZ-P08, CZ-P02/CZ-P03, Hyg–F/Hyg–R and CZ-P04/CZ-P06**		
CZ-P01	GGACATTGGGCTTCATTCAGG	(Cai et al., 2015)
CZ-P07	CCAGCGCCTGCACCAGCTCCTGCCCTCGACATGACACCCCA	This study
GFP-F	GGAGCTGGTGCAGGCGCTGG	This study
GFP-R	GACCGGTTTATTTGTATAG	This study
CZ-P05	GTCTACTGCCGCACCTCAAC	(Cai et al., 2015)
CZ-P08	CTATACAAATAAACCGGTCTAGAGGGTTGACGAGCGTTTAG	This study
CZ-P02	TGTAGAGATACAAGGGAATTCCAGTTGGAGATTGCTACCTCTC	(Cai et al., 2015)
CZ-P03	GTCTGCCCAACGGCTGTTCC	(Cai et al., 2015)
Hyg–F	GAATTCCCTTGTATCTCTACACACAGGC	(Cai et al., 2015)
Hyg-R	GCGCAACTGTTGGGAAGGGCGATC	(Cai et al., 2015)
CZ-P04	CAAGCTTGCATGCCTGCAGGTC	(Cai et al., 2015)
CZ-P06	CCTGAATGATGGCGATGGGA	(Cai et al., 2015)
**Plasmid pCA02 (ΔN): CZ-P06/N-R, CZ-P04/N-F and CZ-P04/CZ-P06**		
N-R	GCCTGCTCGTCGCGGGTAAGCATATTGACGATGAAGGGGC	This study
N-F	CTTACCCGCGACGAGCAGGC	This study
**Plasmid pCA03 (ΔWD2-4):CZ-P06/WD2-4-R, CZ-P04/WD2-4-F and CZ-P04/CZ-P06**		
WD2-4-R	TGGAGGCGGCAGGAAGCGAGGGCCTGCTCGTCGCGGGTAA	This study
WD2-4-F	CTCGCTTCCTGCCGCCTCCA	This study
**Plasmid pCA04 (ΔWD5-6): CZ-P06/WD5-6-R, CZ-P04/WD5-6-F and CZ-P04/CZ-P06**		
WD5-6-R	CCTTGCTCTTCTTCTCGAGAGCGAGCTCCCAAACCTAAA	This study
WD5-6-F	CTCGAGAAGAAGAGCAAGG	This study
**Plasmid pCA05 (ΔC): CZ-P06/C-R,CZ-P04/C-F and CZ-P04/CZ-P06**		
C-R	CCTAAACGCTCGTCAACCCTCTACTTGAGCTCATCAACCTTGC	This study
C-F	TAGAGGGTTGACGAGCGTTTAGG	This study
**Plasmid pCA06 (ΔN): CZ-P01/N-R, CZ-P05/N-F and CZ-S/CZ-A**		
CZ-S	ACCTGCAGGCATGCAAGCTTGTCTGCCCAACGGCTGTTCC	This study
CZ-A	CGACGGCCAGTGCCAAGCTTCAGTTGGAGATTGCTACCTCTC	This study
**Plasmid pCA07 (ΔWD2-4): CZ-P01/WD2-4-R, CZ-P05/WD2-4-F and CZ-S/CZ-A**		
**Plasmid pCA08(ΔWD5-6): CZ-P01/WD5-6-R, CZ-P05/WD5-6-F and CZ-S/CZ-A**		
**Plasmid pCA09 (ΔC): CZ-P01/C-R, CZ-P05/C-F and CZ-S/CZ-A**		
**CpcB point mutations**		
**Plasmid pCA10: CZ-P01/1 G-H-R, CZ-P05/1 G-H-F and CZ-S and CZ-A**		
1 G-H-R	CAGCCATTCTCGGCCTCAAGGGTACCACGAAGAACCATCT	This study
1 G-H-F	CTTGAGGCCGAGAATGGCTG	This study
**Plasmid pCA11: CZ-P01/2 G-H-R, CZ-P05/2 G-H-F and CZ-S and CZ-A**		
2 G-H-R	GATGTGGGACTCAGCCTCGAGGCTGCGCTTGGGGTAACCGTA	This study
2 G-H-F	CTCGAGGCTGAGTCCCACATC	This study
**Plasmid pCA12: CZ-P01/3 G-H-R, CZ-P05/3 G-H-F and CZ-S and CZ-A**		
3 G-H-R	GACGTCGCTGGTCTCGGCAACGAAGGTACGAGTGGTCTCGC	This study

3 G-H-F	GTTGCCGAGACCAGCGACGTC	This study
**Plasmid pCA13: CZ-P01/4 G-H-R, CZ-P05/4 G-H-F and CZ-S and CZ-A**		
4 G-H-R	GAAACCCAGTCGGTCTCGGCCTTGTCGGTGATGGTAAAC	This study
4 G-H-F	GGCCGAGACCGACTGGGTTTC	This study
**Plasmid pCA14: CZ-P01/5 G-H-R, CZ-P05/5 G-H-F and CZ-S and CZ-A**		
5 G-H-R	GATGTAACCGGTCTCAGCGATGTGGTCGGTCTGGAGGCGGCAG	This study
5 G-H-F	CATCGCTGAGACCGGTTACATC	This study
**Plasmid pCA15: CZ-P01/1 W-D-R, CZ-P05/1 W-D-F and CZ-S and CZ-A**		
1 W-D-R	GAGGGACTTCTTCCGAGAGGCAGACAGAGCGTAGGCACCG	This study
1 W-D-F	GCCTCTCGGAAGAAGTCCCTC	This study
**Plasmid pCA16: CZ-P01/1 W-D-R, CZ-P05/1 W-D-F and CZ-S and CZ-A**		
2 W-D-R	CGAGCTTCTTCCGGCCAGCGGAAACGATGACGGGGTTCTG	This study
2 W-D-F	CGCTGGCCGGAAGAAGCTCG	This study
**Plasmid pCA17: CZ-P01/1 W-D-R, CZ-P05/1 W-D-F and CZ-S and CZ-A**		
3 W-D-R	GGACTCGTTGAGCTTCCGGAGCATGGTGGTGCCGTCCTTGCC	This study
3 W-D-F	GCTCCGGAAGCTCAACGAGTCC	This study

Supplementary Table 2. The mRNA fold changes of drug response related genes in the *ΔcpcB* mutant compared with parental wild-type strain

Gene ID	Description	Foldchange	P-value
AFUB_033070	Putative *A. fumigatus erg26*	6.559	0.005
AFUB_071560	Putative orthologs of *S. pombe erg5*	3.212	0.005
AFUB_033030	cellular response to drug	2.460	0.010
AFUB_053630	ABC multidrug transporter *mdr1*	2.351	0.029
AFUB_072040	Putative orthologs of *C. albicans erg5*; *S. pombe erg5*	0.525	0.049
AFUB_068010	Putative orthologs of *S. pombe erg10*	0.357	0.002
AFUB_086300	Putative cytochrome P450 monooxygenase	0.343	0.031
AFUB_033700	P450 monooxygenase and role in steroid metabolic process	0.230	0.000
AFUB_017280	transcript downregulated in response to voriconazole	0.079	0.001
AFUB_033670	P450 monooxygenase; ortholog of *S. pombe erg5*	0.054	2.82E-05

Supplementary Table 3. Genes with significantly differential expression (fold change ≥ 5) in the *ΔcpcB* mutant versus parental wild-type strain in the GO groups

Gene ID	Description	Foldchange	P-value
**metabolic process**			
AFUB_049200	Putative *S. cerevisiae* PMR1 homolog	14.70	1.08E-09
AFUB_054460	Has domain(s) with predicted carbon-sulfur lyase activity	9.62	0.00105104
AFUB_079750	Has domain(s) with predicted oxidoreductase activity	0.19	0.00672334
AFUB_095000	Has domain(s) with predicted catalytic and oxidoreductase activity	0.18	0.00044604
AFUB_090080	Has domain(s) with predicted oxidoreductase activity	0.17	0.00326634
AFUB_018360	*S. cerevisiae* GOR1 homolog, putative	0.15	1.67E-07
AFUB_084950	Protein similar to nonribosomal peptide synthases (NRPS-like)	0.13	0.00328527
AFUB_025250	Ortholog(s) have triglyceride lipase, cutinase activity	0.11	5.11E-05
AFUB_000260	Has domain(s) with predicted oxidoreductase activity	0.07	7.22E-13
**oxidation-reduction process**			
AFUB_079050	Has domain(s) with predicted oxidoreductase activity	27.00	1.84E-05
AFUB_087590	Putative S. cerevisiae ADH3 homolog,	12.40	1.97E-08
AFUB_048750	Putative zinc ion binding, nucleotide binding, oxidoreductase activity	10.50	3.57E-07
AFUB_075790	Ortholog(s) have flavin-linked sulfhydryl oxidase activity	7.70	9.95E-05
AFUB_081320	Putative iron-sulfur cluster-binding protein	7.64	1.37E-08
AFUB_033620	Putative integral membrane protein; transcript induced by voriconazole	7.21	0.00146074
AFUB_033070	Putative C-3 sterol dehydrogenase/C-4 decarboxylase	6.56	0.00486403
AFUB_053780	Putative alcohol dehydrogenase	5.80	5.02E-07
AFUB_084020	Putative glyceraldehyde 3-phosphate dehydrogenase	5.34	3.17E-06
AFUB_015140	Putative zinc-binding dehydrogenase family oxidoreductase	0.19	1.03E-06
AFUB_079750	Has domain(s) with predicted oxidoreductase activity	0.19	0.00672334
AFUB_095000	Putative catalytic activity, oxidoreductase activity	0.18	0.00044604
AFUB_090080	Has domain(s) with predicted oxidoreductase activity	0.17	0.00326634
AFUB_033740	FAD-dependent oxidoreductase	0.17	0.00092097
AFUB_018360	Putative NAD binding, oxidoreductase activity	0.15	1.67E-07
AFUB_017280	Putative catalase with a predicted role in festuclavine biosynthesis	0.08	0.00137322
AFUB_033670	P450 monooxygenase	0.05	2.82E-05
**transmembrane transport**			
AFUB_101840	Putative role in transmembrane transport and localization	16.75	2.11E-14
AFUB_000060	Putativerole in response to stress and localization	15.30	0.00702066
AFUB_049200	*S. cerevisiae* PMR1 homolog, putative	14.73	1.08E-09
AFUB_070460	Putative cation transmembrane transporter activity	6.26	5.06E-07
AFUB_092140	Ortholog(s) have cation ion transmembrane transporter activity	5.38	6.97E-07
AFUB_050080	Putative role in transmembrane transport and localization	0.10	0.00127424
AFUB_044500	Ortholog(s) have role in ferric triacetylfusarinine C transport	0.09	0.00481416
**catalytic activity**			
AFUB_101930	Putative 3-deoxy-7-phosphoheptulonate synthase and catalytic activity	62.67	6.28E-08
AFUB_049200	Putative S. cerevisiae PMR1 homolog	14.73	1.08E-09
AFUB_087620	Has domain(s) with predicted zinc ion binding, hydrolase activity	6.77	5.01E-06

AFUB_095000	Has domain(s) with predicted catalytic activity, oxidoreductase activity	0.18	0.00044604
AFUB_033740	FAD-dependent oxidoreductase	0.17	0.00092097
AFUB_084950	Protein similar to nonribosomal peptide synthases (NRPS-like)	0.13	0.00328527
AFUB_101190	Putative carbon-carbon lyase activity, catalytic activity	0.10	0.00949312
**extracellular region**			
AFUB_080630	Ortholog(s) have extracellular region localization	10.46	6.69E-05
AFUB_075790	Ortholog(s) have flavin-linked sulfhydryl oxidase activity	7.70	9.95E-05
AFUB_084020	Putative glyceraldehyde 3-phosphate dehydrogenase	5.34	3.17E-06
AFUB_068800	Putative secreted alkaline serine protease	0.14	4.74E-06
AFUB_025250		0.11	5.11E-05
**peptidase activity**			
AFUB_068800	Putative secreted alkaline serine protease	0.14	4.74E-06
AFUB_033730	Putative reverse prenyltransferase	0.12	9.17E-10
AFUB_047780	Ortholog(s) have exopeptidase activity	0.10	8.70E-10
**iron ion binding**			
AFUB_081320	Putative iron-sulfur cluster-binding protein	7.64	1.37E-08
AFUB_033620	Putative integral membrane protein	7.21	0.00146074
AFUB_033670	P450 monooxygenase	0.05	2.82E-05
**cation transport**			
AFUB_049200	*S. cerevisiae* PMR1 homolog, putative	14.73	1.08E-09
AFUB_070460	Putative cation transmembrane transporter activity	6.26	5.06E-07
AFUB_092140	Ortholog(s) have cation ion transmembrane transporter activity	5.38	6.97E-07
**uncharacterized function**			
AFUB_081530	Protein of unknown function	27.98	5.14E-06
AFUB_101480	Protein of unknown function	22.45	6.79E-12
AFUB_084940	Protein of unknown function	16.25	1.65E-07
AFUB_101880	Protein of unknown function	14.53	1.62E-13
AFUB_057570	Protein of unknown function	14.47	9.67E-07
AFUB_050510	Protein of unknown function	12.68	1.06E-08
AFUB_078950	Protein of unknown function	11.62	3.21E-06
AFUB_078930	Protein of unknown function	9.72	2.43E-05
AFUB_084100	Protein of unknown function	9.65	1.09E-07
AFUB_091880	Protein of unknown function	8.98	4.48E-05
AFUB_101900	Protein of unknown function	8.93	0.002822581
AFUB_080220	Protein of unknown function	8.82	1.17E-09
AFUB_045320	Protein of unknown function	8.67	5.75E-05
AFUB_028320	Protein of unknown function	8.26	0.000125059
AFUB_049210	Protein of unknown function	7.77	0.000166864
AFUB_048760	Protein of unknown function	7.64	0.000450227
AFUB_003760	Protein of unknown function	7.54	2.81E-06
AFUB_061950	Protein of unknown function	7.29	0.000883611
AFUB_087600	Protein of unknown function	7.29	0.010628358
AFUB_101050	Protein of unknown function	7.29	0.030272893
AFUB_087560	Protein of unknown function	7.15	3.00E-07

AFUB_078200	Protein of unknown function	6.56	0.044453914
AFUB_034130	Protein of unknown function	6.46	9.87E-08
AFUB_080540	Protein of unknown function	6.25	0.006337032
AFUB_028820	Protein of unknown function	6.10	0.010985475
AFUB_069460	Protein of unknown function	6.08	1.49E-07
AFUB_050160	Protein of unknown function	5.99	0.005244576
AFUB_032880	Protein of unknown function	5.69	5.42E-07
AFUB_029430	Protein of unknown function	5.62	0.000281763
AFUB_097090	Protein of unknown function	5.26	1.01E-05
AFUB_034560	Protein of unknown function	5.04	8.81E-06
AFUB_057840	Protein of unknown function	5.04	3.62E-06
AFUB_029950	Protein of unknown function	0.05	3.81E-06
AFUB_077710	Protein of unknown function	0.07	0.028286608
AFUB_094900	Protein of unknown function	0.08	0.002029987
AFUB_035600	Protein of unknown function	0.10	0.024843384
AFUB_025260	Protein of unknown function	0.12	9.70E-07
AFUB_034150	Protein of unknown function	0.13	0.001069581
AFUB_045630	Protein of unknown function	0.13	0.003939245
AFUB_033490	Protein of unknown function	0.15	0.025621322
AFUB_079300	Protein of unknown function	0.15	0.000587316
AFUB_102210	Protein of unknown function	0.16	8.64E-05
AFUB_046180	Protein of unknown function	0.17	1.25E-06
AFUB_046170	Protein of unknown function	0.17	4.26E-07
AFUB_017210	Protein of unknown function	0.18	0.031364757
AFUB_033750	Protein of unknown function	0.19	5.61E-06
AFUB_019550	Protein of unknown function	0.20	0.01785142

References

Cai, Z.D., Chai, Y.F., Zhang, C.Y., Qiao, W.R., Sang, H., and Lu, L. (2015). The G beta-like protein CpcB is required for hyphal growth, conidiophore morphology and pathogenicity in *Aspergillus fumigatus*. *Fungal Genetics and Biology* 81**,** 120-131. doi: 10.1016/j.fgb.2015.04.007.

Gouet, P., Courcelle, E., and Stuart, D.I. (1999). ESPript: analysis of multiple sequence alignments in PostScript. *Bioinformatics* 15**,** 305-308. doi: 10.1093/bioinformatics/15.4.305.

Thompson, J.D., Gibson, T.J., Plewniak, F., Jeanmougin, F., and Higgins, D.G. (1997). The CLUSTAL_X windows interface: flexible strategies for multiple sequence alignment aided by quality analysis tools. *Nucleic acids research* 25**,** 4876-4882. doi: 10.1093/nar/25.24.4876.
